# Network Pharmacology and Bioinformatics Analysis to Identify the Molecular Targets and its Biological Mechanisms of Sciadopitysin against Glioblastoma

**DOI:** 10.7150/jca.94202

**Published:** 2024-05-13

**Authors:** Haiwei Lian, Yajie Xiong, Guojie Zhao, Meng Yi, Jingchao Wang, Huimin Liu, Yun Zhou

**Affiliations:** 1 Department of Neurosurgery, Renmin Hospital of Wuhan University, Wuhan, Hubei, 430060, P.R. China.; 2 Department of Basic Medicine, Medical School, Kunming University of Science and Technology, Kunming, Yunnan, 651701, P.R. China.; 3 Guangdong Key Laboratory of Genome Instability and Human Disease Prevention, Department of Biochemistry and Molecular Biology, School of Medicine, Shenzhen University, Shenzhen, Guangdong, 518055, P.R. China.; 4 Department of pediatrics, Liyuan Hospital, Tongji Medical College, Huazhong University of Science and Technology, Wuhan, Hubei, 430077, P.R. China.; 5 Department of Gynaecology and Obstetrics, Renmin Hospital of Wuhan University, Wuhan, Hubei, 430060, P.R. China.

**Keywords:** sciadopitysin, glioblastoma, network pharmacology, HSP90, AKT1

## Abstract

Glioblastoma multiform (GBM) is categorized as the most malignant subtype of gliomas, which comprise nearly 75% of malignant brain tumors in adults. Increasing evidence suggests that network pharmacology will be a novel method for identifying the systemic mechanism of therapeutic compounds in diseases like cancer. The present study aimed to use a network pharmacology approach to establish the predictive targets of sciadopitysin against GBM and elucidate its biological mechanisms. Firstly, targets of sciadopitysin were obtained from the SwissTargetPrediction database, and genes associated with the pathogenesis of GBM were identified from the DiGeNET database. Sixty-four correlative hits were identified as anti-glioblastoma targets of sciadopitysin. Functional enrichment and pathway analysis revealed significant biological mechanisms of the targets. Interaction of protein network and cluster analysis using STRING resulted in two crucial interacting hub genes, namely, HSP90 and AKT1. Additionally, the *in vitro* cytotoxic potential of sciadopitysin was assessed on GBM U87 cells. The findings indicate that the pharmacological action of sciadopitysin against GBM might be associated with the regulation of two core targets: HSP90 and AKT1. Thus, the network pharmacology undertaken in the current study established the core active targets of sciadopitysin, which may be extensively applied with further validations for treatment in GBM.

## Introduction

Adult glioblastoma (GBM) is the most common malignant primary brain tumor, representing approximately 57% of all gliomas and 48% of all primary malignant central nervous system (CNS) tumors 1. It is also one of the most deadly and recalcitrant of all malignant solid tumors. Despite recent advances that have been made in multimodality therapy for glioblastoma incorporating surgery, radiotherapy, systemic therapy (chemotherapy, targeted therapy), and supportive care, the overall prognosis is still poor, and long-term survival is rare. The latest research shows that in the United States alone, the annual incidence of glioblastoma is about 34 cases per 1 million people, with a median survival of about 8 months, and more than 7,000 people die from glioblastoma each year 2. For most patients with GBM, there is no known cause of the disease. It may occur at any age and originate by genetic alterations affecting neuroglial stem or progenitor cells 3. Incidence increases steadily with age. The therapeutic efficacy of glioblastoma could be improved by figuring out molecular pathways and alterations in the signaling mechanisms of the tumor cells.

Sciadopitysin (SP), a biflavonoid compound that is common in gymnosperms such as *Cyperus roxburghii, Cryptomeria fortunei*, *Podocarpus*, *Taxus chinensis*, and *Ginkgo biloba*, exhibits various biological properties 4. In recent years, the biological activity of SP has been gradually investigated. Studies have shown that SP has various pharmacological effects, such as anti-tumor, antioxidation, reducing blood glucose and blood lipid, etc. Bioflavonoids show potential proteins or enzymes related to metabolism, growth, and survival, which are related to tumor growth, tumor metastasis, and angiogenesis 5. Glioblastoma is a kind of heterogeneous disease, meaning a distinct understanding of its mechanism is required for significant treatment preferences. However, due to limitations in research techniques and economic considerations, it is difficult to reveal the synergy between multiple targets in disease treatment. Currently, the precise antitumor mechanisms of SP are unknown.

Network pharmacology, as an emerging discipline, has been developed very well in recent years by integrating bioinformatics and pharmacology. It is a kind of method that predicts targets against a particular disease by the available biomedical data in system biology and poly-pharmacology 6. By establishing a pharmaceutical chemistry database, researchers can discover the relationship between drugs and targets, targets and target diseases, systematically mine the existing biological data, abstract these data into a network relation model, and then systematically explain the role of drugs in disease treatment 7. Network pharmacology can describe the complex relationship between biological systems in targeted therapy and determine the synergistic effect in tumor therapy through network component analysis 8.

So, we used network pharmacology to study multi-target drugs, exploring target sites and action pathways to provide evidence for the clinical application of SP in GBM treatment. The study was divided into the following stages: (1) Identification of the potential targets of SP based on its association with GBM through retrieval from databases; (2) Using gene ontology (GO) terms to study the key role of identified targets through functional enrichment and pathway analysis; (3) Determining the core indicators based on interaction through network analysis; (4) Validation of potential targets by molecular docking verification and *in vitro* assessment.

## Materials and Methods

### Identification of potential targets of sciadopitysin and glioblastoma-related targets

SwissTargetPrediction (http://www.swisstargetprediction.ch/, accessed on 20 July 2023) was utilized to screen the potential targets of sciadopitysin 9. The molecular structure is displayed in Figure S1, and SMILES of sciadopitysin is COC1=CC=C(C=C1)C2OC3C(=C(O)C=C(O)C=3C(=O)C=2)C4=C(OC)C=CC(=C4)C5OC6C(=C(O)C=C(OC)C=6)C(=O)C=5.

The glioblastoma-related targets were screened from the DisGeNET database (http://www.disgenet.org/, accessed on 20 July 2023) using the keyword search “glioblastoma multiforme” 10. The overlapped targets of sciadopitysin potential targets and glioblastoma-related targets were considered as candidate anti-glioblastoma cancer sciadopitysin targets, which were subjected to further analysis.

### Gene ontology and signaling pathway enrichment analysis of the drug-disease targets

To investigate the biological characteristics of drug-disease targets, Gene Ontology (GO) enrichment, and Kyoto Encyclopedia of Genes and Genome (KEGG) pathway analyses were carried out using the clusterProfiler package. The enrichment terms with adjusted *p*-value <0.05 were deemed to be significantly different. The genes with significant regulatory pathways were chosen for subsequent gene-pathway network analysis.

### Establishment of the PPI network and module construction of glioblastoma targets

Based on the Search Tool for the Retrieval of Interacting Genes (STRING), the protein-protein interaction network was constructed by the candidate 64 anti-glioblastoma targets of sciadopitysin, with a lowest confidence score of 0.4.

### Network construction and analysis

To elucidate the therapeutic mechanism of sciadopitysin on GBM, the ingredient-target network was built through Cytoscape 3.7.2 software. The drug-disease targets' protein-protein interaction (PPI) network was established by the plugin Bisogenet of Cytoscape version 3.7.2 11. Subsequently, topology analysis was conducted using the Cytoscape plugin CytoNCA to calculate the Betweenness Centrality (BC) and Degree Centrality (DC). The nodes with BC and DC were identified as the key nodes. Next, the gene-pathway network was built to identify the key target genes responsible for sciadopitysin-treated GBM. Lastly, the crossover genes between the key nodes and the key target genes were considered the core molecular targets of sciadopitysin for treating GBM and were selected to perform further *in vitro* experiments.

### Molecular docking analysis

To assess the putative targets identified through network analysis, we conducted a molecular docking analysis using the Auto-Dock Vina software 12. Initially, the three-dimensional (3D) structure of sciadopitysin was retrieved from PubChem (accessed on 9 October 2023) and optimized. Additionally, the 3D structures of the potential targets were downloaded from the Protein Data Bank (PDB) database (accessed on 9 October 2023). We then utilized AutoDockTools to generate a two-dimensional map of sciadopitysin with the potential targets, which enabled us to visualize the direct interactions between the compound and the targets. Finally, PyMol software was used to observe the three-dimensional structure of sciadopitysin-target complex 13.

### Cell and cell culture

Human glioblastoma cell line U87 was purchased from the Chinese Academy of Sciences Cell Bank. These cells were incubated at 37°C, 5% CO2 concentration, and high humidity. They were regularly cultured in Dulbecco's modified Eagle's medium (HyClone), which were supplemented with 10 % fetal bovine serum (HyClone).

### Active cell apoptosis

Cell apoptosis was measured by the Caspase 3/7Activity Assay Kit (Absin, China). In brief, cells were collected and centrifuged at 600 g at 4°C for 5 minutes, the supernatant was removed, and after washing with PBS, the lysate was added at a ratio of 100 microliters per 2 million cells. The lysate was dissolved in an ice bath for 15 minutes. Then we centrifuged at 16,000 g and 4°C for 10 minutes and transferred the supernatant to a centrifuge tube pre-cooled by an ice bath. The reaction system was as follows: 40 µL buffer, 50 µL experimental cells, 10 µL Ac-DEVD-pNA (2mM). After incubation at 37°C for one hour, A405 was determined when the color change was noticeable.

### Flow cytometry

The apoptosis of cells was detected by the Annexin V-fluorescein isothiocyanate/propidium iodide (Annexin V-FITC/PI) apoptosis detection kit (abs50001; Absin Biotechnology Co. Ltd., Shanghai, China) staining. U87 cells were incubated in 6-well plates and treated with 100 μΜ sciadopitysin for 72 h. Cells were collected and washed with cold phosphate-buffered brine, resuspended with 5 µL Annexin V-FITC and 5 µL PI staining solution in 300 µL 1×binding buffer, and incubated at room temperature for 15 min under darkness. Next, we added 300 µL 1×binding buffer and mixed. Finally, cell apoptosis was detected by flow cytometry (Bio-Rad, State of California, USA).

### Western blot

Total protein extraction of cell lines was conducted by applying RIPA buffer (Beyotime, Shanghai, China). The proteins were separated on a 10-12% sodium dodecyl sulfate-polyacrylamide gel and then transferred onto polyvinylidene fluoride membranes (Millipore, Billerica, MA, USA). Next, the membrane was incubated with primary antibodies overnight at 4°C. AKT1 (1:1000, rabbit, Cell Signaling Technology, Cat#75692), HSP90α (1:1000, mouse, Abcam, Cat#ab79849), Actin (1:1000, rabbit, Abcam, Cat# ab179467). After the membrane was washed, HRP-labeled Goat Anti-Rabbit IgG (1:1000, Beyotime, Cat# A0208, RRID: AB_2892644) or HRP-labeled Goat anti-mouse IgG (1:1000, Beyotime, A0216, RRID: AB_2860575) were incubated at 25°C for 1 hour. Finally, the enhanced chemiluminescence detection system (Applygen Technology, Beijing, China) was used to detect the signal. The protein expression level was detected by ImageJ software.

### Statistical analysis

SPSS version 23.0 was employed to conduct the statistical test. All experiments were performed in triplicate. All data were presented as mean ± SD. Comparisons among different groups were done with one-way ANOVA followed by Dunnett or Bonferroni post hoc analysis. A *p*-value of <0.01 or <0.05 was deemed statistically significant. The statistical column charts were drawn with Prism 8.0.

## Results

### Prediction and screening of drug target proteins and glioblastoma-related targets

**Figure 1:**
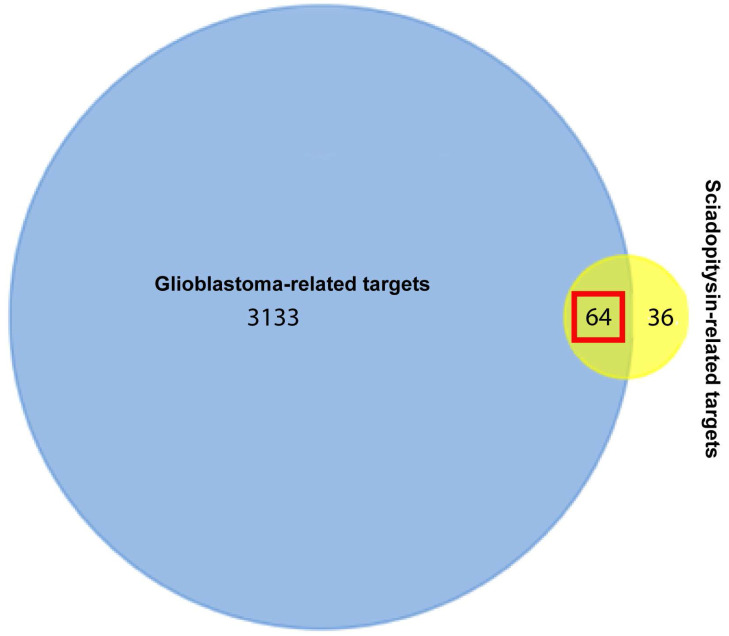
Venn diagram showing the 64 overlapped targets of sciadopitysin potential targets and glioblastoma-related targets.

One hundred potential targets of sciadopitysin were screened from the SwissTargetPrediction database. The classes of these potential targets were primarily kinase, enzyme, oxidoreductase (Figure S2). 3197 hits of glioblastoma-related targets were predicted from the DisGeNET database. There were 64 targets overlapped in the drug and disease targets (Figure 1 and Table 1).

### Gene ontology analysis of the candidate targets

**Figure 2:**
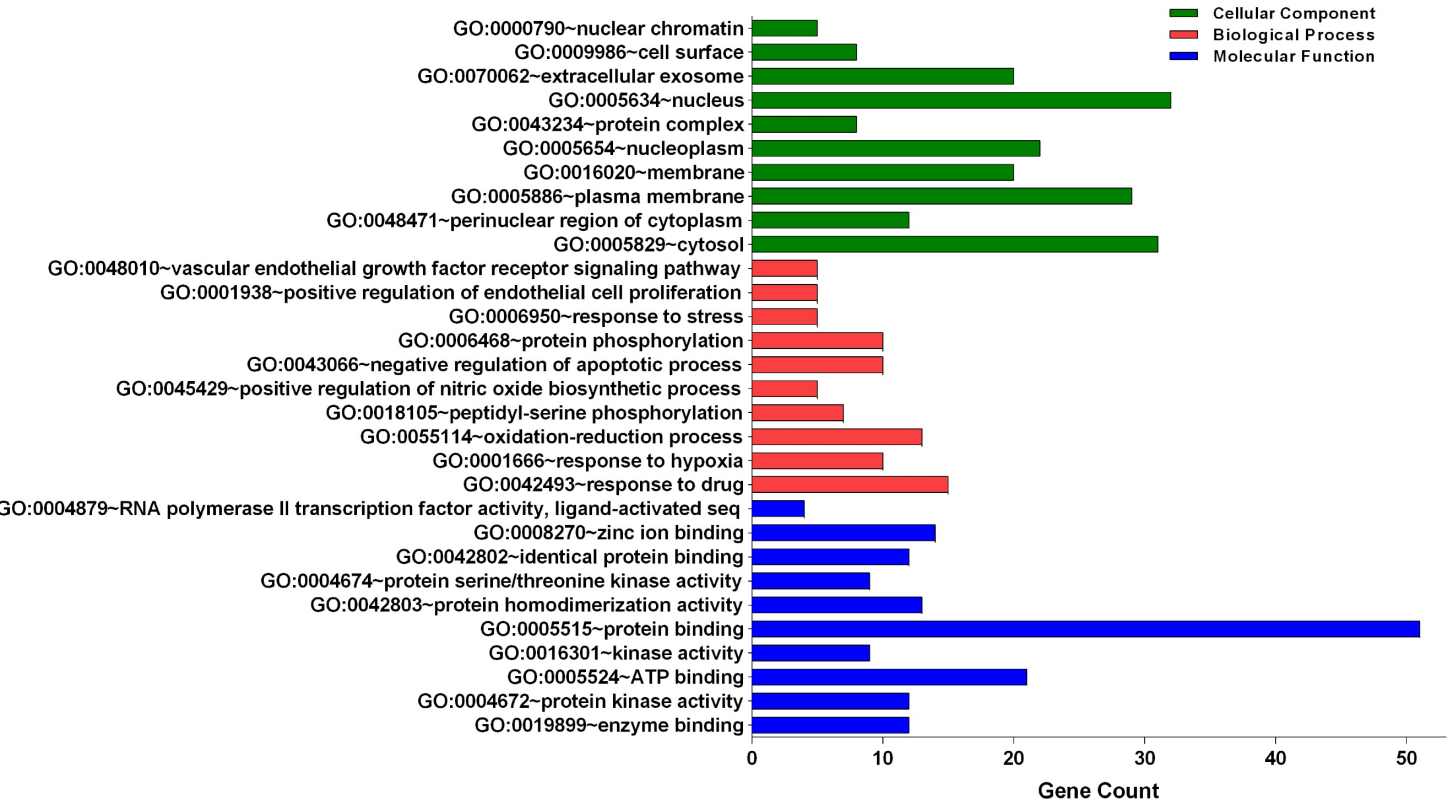
Gene Ontology analysis of candidate targets. GO analysis classified into 3 groups: molecular function, biological process and cellular component.

Three categories of anti-glioblastoma targets of sciadopitysin: cellular component, biological process, and molecular function, were classified by gene ontology (GO) analysis (Figure 2). The top 10 significantly enriched GO terms among the 64 core targets were listed in Tables 2-4. Among these GO functions, the enriched GO terms of cellular component were nucleus (GO:0005634), cytosol (GO:0005829), and plasma membrane (GO:0005886). The significant GO terms of the biological process included a response to drug (GO:0042493) and oxidation-reduction process (GO:0055114). The enriched GO terms of molecular function were associated with protein binding (GO:0005515) and ATP binding (GO:0005524).

### Signaling pathway enrichment analysis of the candidate targets

**Figure 3:**
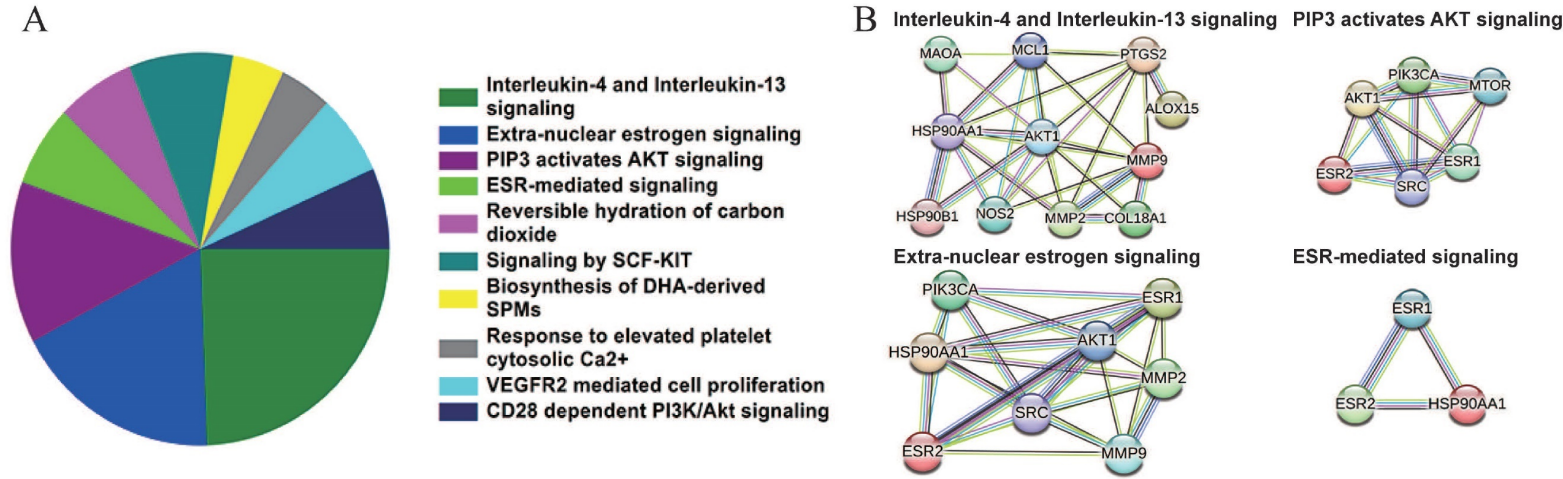
Signaling pathway enrichment analysis of the candidate targets of sciadopitysin. (A) Pie chart shows the top 10 enriched pathways of candidate targets of sciadopitysin identified by Reactome database. (B) The four most significant enriched pathways are based on the protein-protein interaction network.

Based on the Reactome database, the top 10 enriched pathways of the 64 candidate targets were found to be Interleukin-4 and Interleukin-13 signaling, Extra-nuclear estrogen signaling, PIP3 activates AKT signaling, ESR-mediated signaling, Reversible hydration of carbon dioxide, Signaling by SCF-KIT, Response to elevated platelet cytosolic Ca2+, Biosynthesis of DHA-derived SPMs, VEGFR2 mediated cell proliferation and CD28 dependent PI3K/Akt signaling (Figure 3A and Table 5). Based on the interaction network constructed by the STRING database, the targets among the pathway enrichment were grouped into four most significant clusters that include ESR-mediated signaling string, extra-nuclear estrogen signaling string, interleukin-4 and Interleukin-13 signaling string, and PIP3 activates AKT signaling string, respectively (Figure 3B).

### PPI network analysis

**Figure 4:**
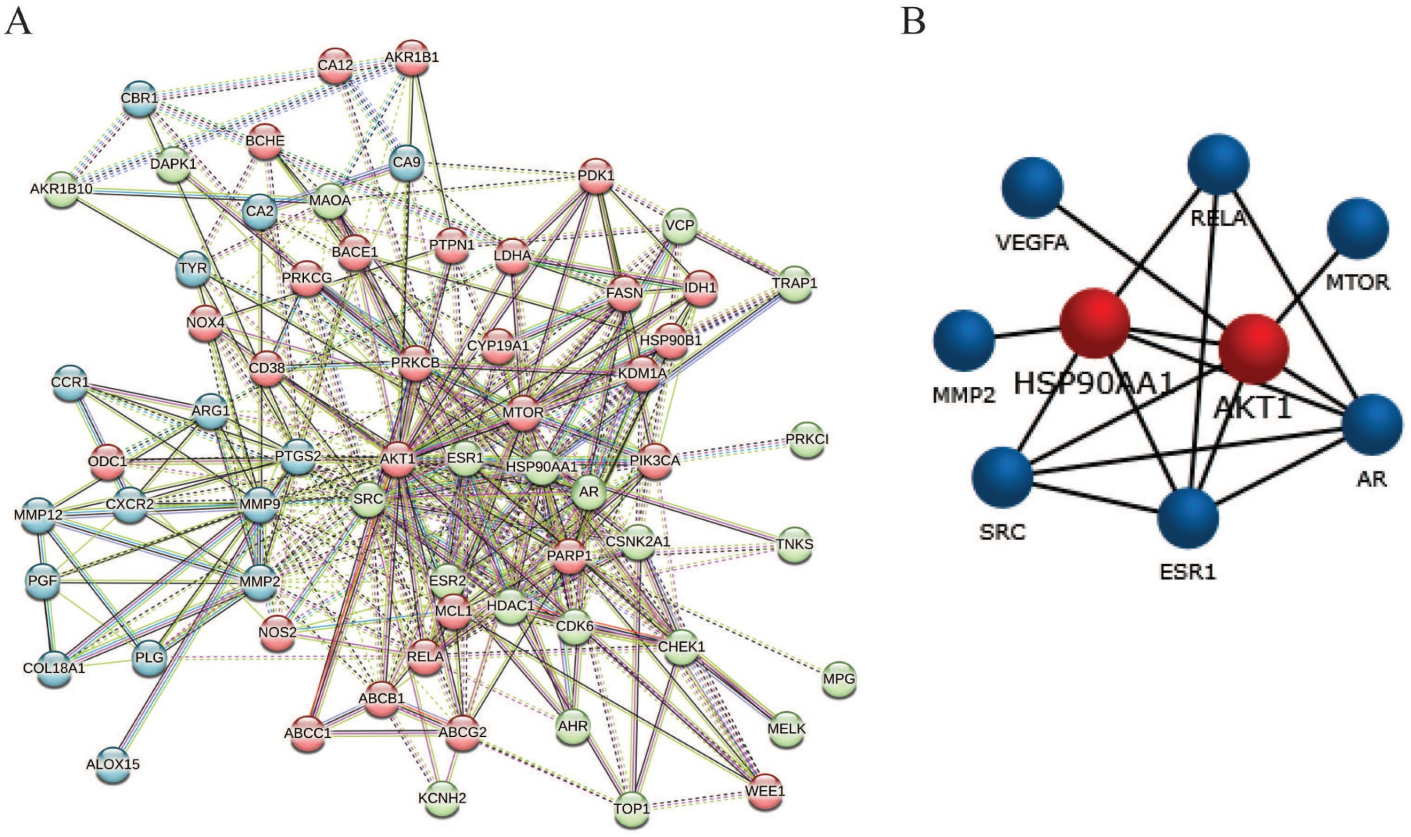
Protein-protein interaction network complex and sub-network module construction of candidate targets. (A) Protein-protein interaction network of the anti-glioblastoma targets of sciadopitysin. A total of 64 targets was screened, containing 64 nodes and 359 edges. (B) One key sub-network of the core targets was constructed by module analysis.

The candidate 64 anti-glioblastoma targets of sciadopitysin were introduced into the STRING database to obtain the PPI network complex. The PPI network contained 64 nodes and 359 edges (Figure 4A), obtained with a medium confidence score of 0.4 and enriched *p*-value of < 1.0e-16. Using CFinder with a k-cliques value of >12, one module (Figure 4B) was extracted from the constructed PPI network. Based on the key module, HSP90AA1 and AKT1 were selected as the key genes of sciadopitysin targets in glioblastoma.

### Molecular docking

**Figure 5:**
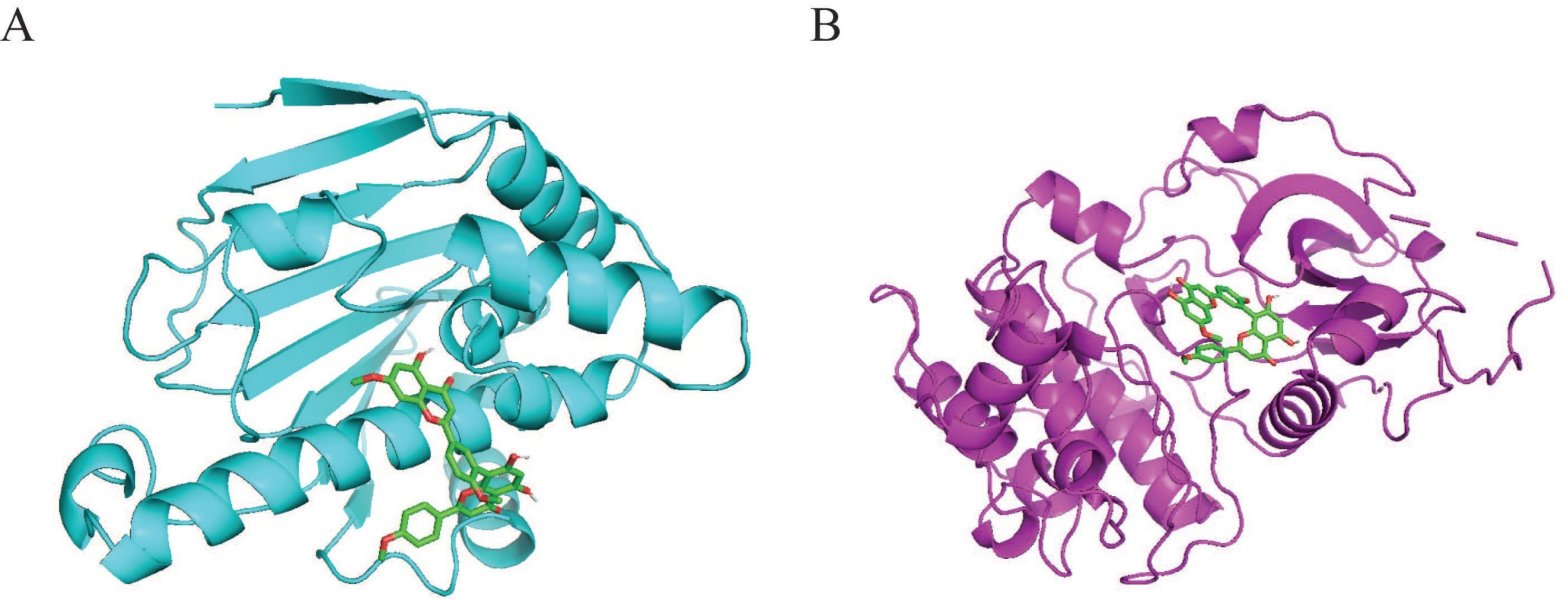
Interaction of sciadopitysin with HSP90α (PDB ID:2VCJ) and AKT1 (PDB ID:4EKL). (A) 3D docking molecule of HSP90α (cyan) and sciadopitysin. (B) 3D docking molecule of AKT1 (magenta) and sciadopitysin.

Molecular docking was performed to validate the possible binding action mode between sciadopitysin and core targets. Among the two targets, the free binding energy of sciadopitysin with HSP90α -8.1kcal/mol, and with AKT1 was -9.3 kcal/mol, shown in Table 6. In the visualized image, it can be clearly seen that there are docking groups between the cyan labeled HSP90α and sciadopitysin molecules. And magenta-labeled AKT1 and sciadopitysin molecules are identical (Figure 5). The docking diagram shows that HSP90α and AKT1 both interact directly with sciadopitysin.

### Sciadopitysin promoted apoptosis of GBM cells by inhibiting HSP90a and AKT1

**Figure 6:**
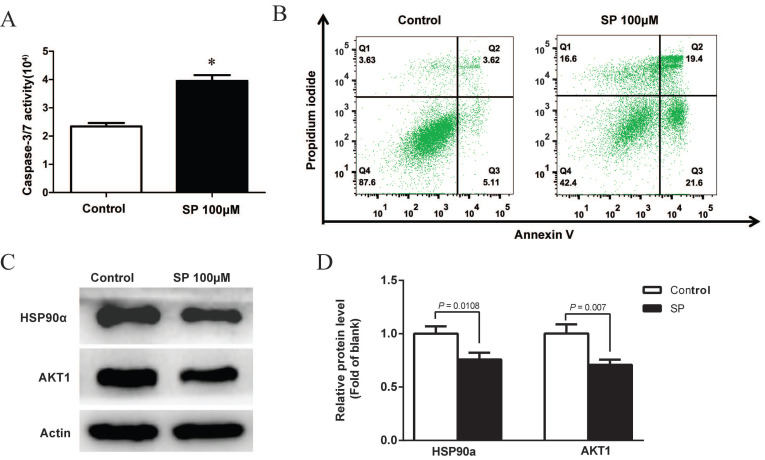
Sciadopitysin promoted apoptosis of U87 cells. (A) Apoptosis of U87 cells was detected by measuring the activity of Caspase 3/7. (B) Apoptosis was analyzed by flow cytometry after Annexin V-FITC/PI staining. (C) Representative Western blots showing the status of HSP90α, AKT1 in U87 cells. Actin was used as an internal control. (D) The expression levels of HSP90α and AKT1 proteins were statistically analyzed.

After treatment with 100µM sciadopitysin for 72 h, the apoptosis of GBM cells was determined by flow cytometry, and results showed that the percentage of apoptotic cells was significantly increased after sciadopitysin treatment (Figure 6B). These results strongly suggest that sciadopitysin induces apoptosis of GBM cells. We further verified the molecular target of sciadopitysin involved in inducing apoptosis in U87 cells. According to the above analysis, we detected the HSP90α and AKT1 proteins through western blotting. As shown in Figures 6C and 6D, compared with the control group, the protein expression levels of HSP90α and AKT1 in the treatment group were significantly decreased. The results confirmed that HSP90α and AKT1 were the molecular targets of sciadopitysin in inhibiting glioblastoma.

## Discussion

In this study, bioinformatics investigation and network pharmacology were used to investigate the possibility of sciadopitysin in treating glioblastoma. To our knowledge, this is the first study to combine network pharmacology and molecular docking simulations to reveal the anti-glioblastoma effects of sciadopitysin. In the study, firstly, GBM-related genes were predicted from the public database prediction database, the target of sciadopitysin was found, and 64 targets of sciadopitysin anti-glioblastoma were obtained. PPI network and STRING cluster analysis identified HSP90α and AKT1 as two key hubs of action. Finally, cell experiments confirmed that sciadopitysin could regulate GBM apoptosis through HSP90α and AKT1.

HSP90α, encoded by the HSP90AA1 gene, is an important chaperone protein that requires a variety of collaborators to function 14. And the HSP90 protein plays an important role in basic cellular processes and regulatory pathways such as apoptosis, cell cycle control, and cell signaling 15. Overexpression of HSP90 is strongly associated with various cancers; for example, in breast cancer, the expression level of HSP90 is closely related to the survival of patients, and the abnormally high expression level of HSP90 reflects the poor treatment effect 16-18. HSP90 is a promising marker for the diagnosis and prognosis of malignant tumors 14, 19. In glioblastoma, the expression of HSP90α was abnormally high 20, 21. Research shows the inhibitor of HSP90, 17-Allylamino-17-deoxykygdanamycin (17-AAG), inhibits the growth of glioma cell lines by targeting intracellular EGFR, AKT, and MAPK proteins 22. In addition, studies have also shown that inhibiting Hsp90 function in glioblastoma cell lines can reduce the expression level of cell division cycle 2 kinase (cdc2) and cell division cycle 25c (cdc25c), and the proliferation is blocked in G(2)/M 23. In our study, sciadopitysin promotes apoptosis of U87 cells, which is closely related to the HSP90α protein. In normal tissues, HSP90α is present in the cytoplasm, whereas in glioma cell lines, HSP90α may be abnormally localized to the cell membrane 19, 24. Sciadopitysin interacts with HSP90α to reduce the activity of intracellular cdc2 and cdc25c and block the cell cycle, thereby inducing apoptosis.

AKT is one of the main downstream effect targets of phosphatidylinositol 3-kinase, which is overexpressed and activated in various cancers, including glioblastoma 25. The individual functions of the three isomers of AKT, AKT1, AKT2, and AKT3, remain controversial in GBM. AKT2 mRNA and protein levels are elevated in malignant gliomas, while AKT3 expression levels are decreased 26. In glioma cell lines, AKT2 or AKT3 knockdown inhibited cell growth and induced apoptosis. In contrast, AKT1 knockdown did not affect cell growth and apoptosis, suggesting that AKT2 and AKT3 may be the main contributors to GBM cell growth, and AKT1 may be unnecessary 26. However, many studies have shown that AKT1 plays an important role in glioblastoma. For example, AKT1 is one of the key hub genes in the gene network of glioblastoma 27. Overacting insulin receptor substrate 1 may promote GBM cell viability through AKT1 activation 28. The inhibitory effect of SOX4 on the growth of GBM cells is related to the activation of the p53-p21 signal and the decrease of AKT1 activity 29. In our experiment, AKT1 is another key target of sciadopitysin to promote apoptosis of glioma cell lines. After sciadopitysin treatment, AKT1 activity is reduced in U87 cells, which may lead to decreased expression levels of Cyclin D1 or p53 in the cells, thus affecting the cell cycle of glioblastoma.

In conclusion, sciadopitysin could be one novel potential targeted medicine for malignant glioblastoma. HSP90α and AKT1 were the key targets that sciadopitysin plays anti-tumor effects. This study provides preliminary experimental evidence of sciadopitysin against glioblastoma to some extent; However, further research is needed to provide more experimental verifications including HSP90α and AKT1 downstream signaling cascades and animal experiments to explore *in vivo* anti-tumor effects of sciadopitysin.

## Supplementary Material

Supplementary figures.

Raw data.

## Figures and Tables

**Table 1:** List of 64 potential anti‐glioblastoma targets of sciadopitysin.

Gene Name	Uniprot ID	Protein Name
BACE1	P56817	Beta-site APP cleaving enzyme 1
PTPN1	P18031	Tyrosine-protein phosphatase non-receptor type 1
VCP	P55072	Valosin-containing protein
PGF	P49763	Placenta growth factor
VEGFA	P15692	Vascular endothelial growth factor A
ABCG2	Q9UNQ0	ATP-binding cassette sub-family G member 2
MCL1	Q07820	Induced myeloid leukemia cell differentiation protein Mcl-1
ABCB1	P08183	ATP-dependent translocase ABCB1
AKR1B1	P15121	Aldo-keto reductase family 1 member B1
PRKCB	P05771	Protein kinase C beta type
CYP19A1	P11511	Cytochrome P450 19A1
NOX4	Q9NPH5	NADPH oxidase 4
KDM1A	O60341	Lysine-specific histone demethylase 1A
RELA	Q04206	Transcription factor p65
PTGS2	P35354	Prostaglandin G/H synthase 2
TNKS	O95271	Poly [ADP-ribose] polymerase tankyrase-1
ESR1	P03372	Estrogen receptor (ER) (ER-alpha)
PRKCG	P05129	Protein kinase C gamma type (PKC-gamma)
ABCC1	P33527	Multidrug resistance-associated protein 1
ODC1	P11926	Ornithine decarboxylase (ODC)
LDHA	P00338	L-lactate dehydrogenase A chain (LDH-A)
IDH1	O75874	Isocitrate dehydrogenase [NADP] cytoplasmic (IDH)
HSP90AA1	P07900	Heat shock protein HSP 90-alpha
FASN	P49327	Fatty acid synthase
BCHE	P06276	Cholinesterase
MAOA	P21397	Amine oxidase [flavin-containing] A
ESR2	Q92731	Estrogen receptor beta (ER-beta)
CDK6	Q00534	Cyclin-dependent kinase 6
CSNK2A1	P68400	Casein kinase II subunit alpha (CK II alpha)
AKR1B10	O60218	Aldo-keto reductase family 1 member B10
MPG	P29372	DNA-3-methyladenine glycosylase
MELK	Q14680	Maternal embryonic leucine zipper kinase (hMELK)
CA2	P00918	Carbonic anhydrase 2
CA12	O43570	Carbonic anhydrase 12
KCNH2	Q12809	Potassium voltage-gated channel subfamily H member 2
CCR1	P32246	C-C chemokine receptor type 1
TYR	P14679	Tyrosinase
AHR	P35869	Aryl hydrocarbon receptor
PDK1	Q15118	[Pyruvate dehydrogenase (acetyl-transferring)] kinase isozyme 1, mitochondrial
NOS2	P35228	Nitric oxide synthase, inducible
TRAP1	Q12931	Heat shock protein 75 kDa, mitochondrial
AKT1	P31749	RAC-alpha serine/threonine-protein kinase (Protein kinase B alpha)
SRC	P12931	Proto-oncogene tyrosine-protein kinase Src
DAPK1	P53355	Death-associated protein kinase 1
MTOR	P42345	Serine/threonine-protein kinase mTOR
PIK3CA	P42336	Phosphatidylinositol 4,5-bisphosphate 3-kinase catalytic subunit alpha isoform
CHEK1	O14757	Serine/threonine-protein kinase Chk1
WEE1	P30291	Wee1-like protein kinase
CA9	Q16790	Carbonic anhydrase 9
PLG	P00747	Plasminogen
HDAC1	Q13547	Histone deacetylase 1
AR	P10275	Androgen receptor
CBR1	P16152	Carbonyl reductase [NADPH] 1
HSP90B1	P14625	Endoplasmin (Heat shock protein 90 kDa beta member 1)
CXCR2	P25025	C-X-C chemokine receptor type 2
ALOX15	P16050	Polyunsaturated fatty acid lipoxygenase ALOX15
PARP1	P09874	Poly [ADP-ribose] polymerase 1
MMP9	P14780	Matrix metalloproteinase-9
MMP2	P08253	72 kDa type IV collagenase
MMP12	P39900	Macrophage metalloelastase
CD38	P28907	ADP-ribosyl cyclase/cyclic ADP-ribose hydrolase 1
TOP1	P11387	DNA topoisomerase 1
ARG1	P05089	Arginase-1
PRKCI	P41743	Protein kinase C iota type

**Table 2:** Top 10 significantly enriched GO terms of cellular component associated with the identified anti‐glioblastoma targets of sciadopitysin

Term Name	Description	Count	% of Genes	Fold Enrichment	*p*-value
GO:0005829	cytosol	31	48.4375	2.66282051	8.72E-08
GO:0048471	perinuclear region of cytoplasm	12	18.75	5.50241546	8.09E-06
GO:0005886	plasma membrane	29	45.3125	2.00382189	9.85E-05
GO:0016020	membrane	20	31.25	2.58863636	1.05E-04
GO:0005654	nucleoplasm	22	34.375	2.2501796	2.80E-04
GO:0043234	protein complex	8	12.5	5.52912621	5.33E-04
GO:0005634	nucleus	32	50	1.68273315	8.74E-04
GO:0070062	extracellular exosome	20	31.25	2.02596941	0.00232391
GO:0009986	cell surface	8	12.5	4.20295203	0.00259475
GO:0000790	nuclear chromatin	5	7.8125	7.37694301	0.00446334

**Table 3:** Top 10 significantly enriched GO terms of biological process associated with the identified anti‐glioblastoma targets of sciadopitysin

Term Name	Description	Count	% of Genes	Fold Enrichment	*p*-value
GO:0042493	response to drug	15	23.4375	12.9461349	5.08E-12
GO:0001666	response to hypoxia	10	15.625	15.2543605	1.48E-08
GO:0055114	oxidation-reduction process	13	20.3125	5.76161318	1.70E-06
GO:0018105	peptidyl-serine phosphorylation	7	10.9375	14.693	7.24E-06
GO:0045429	positive regulation of nitric oxide biosynthetic process	5	7.8125	30.5087209	1.99E-05
GO:0043066	negative regulation of apoptotic process	10	15.625	5.76648352	4.70E-05
GO:0006468	protein phosphorylation	10	15.625	5.75383772	4.78E-05
GO:0006950	response to stress	5	7.8125	21.5061475	8.00E-05
GO:0001938	positive regulation of endothelial cell proliferation	5	7.8125	19.0126812	1.30E-04
GO:0048010	vascular endothelial growth factor receptor signaling pathway	5	7.8125	18.2204861	1.53E-04

**Table 4:** Top 10 significantly enriched GO terms of molecular function associated with the identified anti‐glioblastoma targets of sciadopitysin

Term Name	Description	Count	% of Genes	Fold Enrichment	*p*-value
GO:0019899	enzyme binding	12	18.75	9.505067568	3.68E-08
GO:0004672	protein kinase activity	12	18.75	8.816678273	7.91E-08
GO:0005524	ATP binding	21	32.8125	3.70506898	2.55E-07
GO:0016301	kinase activity	9	14.0625	9.850168568	3.02E-06
GO:0005515	protein binding	51	79.6875	1.531251779	6.43E-06
GO:0042803	protein homodimerization activity	13	20.3125	4.697196062	1.37E-05
GO:0004674	protein serine/threonine kinase activity	9	14.0625	6.313538896	7.47E-05
GO:0042802	identical protein binding	12	18.75	4.225884513	9.10E-05
GO:0008270	zinc ion binding	14	21.875	3.15886976	3.18E-04
GO:0004879	RNA polymerase II transcription factor activity, ligand-activated sequence-specific DNA binding	4	6.25	29.30729167	3.24E-04

**Table 5:** Top 10 significantly enriched pathways identified by Reactome database and associated anti‐glioblastoma targets of sciadopitysin

Pathway name	Count	*p*-value	Genes
Interleukin-4 and Interleukin-13 signaling	11	2.98418E-11	VEGFA; MCL1; PTGS2; HSP90AA1; MAOA; NOS2; AKT1; HSP90B1; ALOX15; MMP9; MMP2;
Extra-nuclear estrogen signaling	8	9.14128E-10	ESR1; HSP90AA1; ESR2; AKT1; SRC; PIK3CA; MMP9; MMP2;
PIP3 activates AKT signaling	6	1.82899E-05	ESR1; ESR2; AKT1; SRC; MTOR; PIK3CA;
ESR-mediated signaling	3	2.95625E-05	ESR1; HSP90AA1; ESR2;
Reversible hydration of carbon dioxide	3	3.92538E-05	CA2; CA12; CA9;
Signaling by SCF-KIT	4	5.84809E-05	SRC; PIK3CA; CHEK1; MMP9;
Response to elevated platelet cytosolic Ca2+	2	9.86843E-05	PRKCB; PRKCG;
Biosynthesis of DHA-derived SPMs	2	9.86843E-05	PTGS2; ALOX15;
VEGFR2 mediated cell proliferation	3	0.00016796	VEGFA; PRKCB; SRC;
CD28 dependent PI3K/Akt signaling	3	0.000263645	AKT1; MTOR; PIK3CA;

**Table 6:** Molecular docking studies of sciadopitysin with target protein and their binding energies

Target	drug	Binding energy/(kcal/mol)
HSP90α	Sciadopitysin	-8.1
AKT1	Sciadopitysin	-9.3
