# Supplementary material for: Network Pharmacology and Bioinformatics Analysis to Identify the Molecular Targets and its Biological Mechanisms of Sciadopitysin against Glioblastoma
Source: J Cancer. 2024 May 13;15(12):3675–83. doi: 10.7150/jca.94202 (PMC11190769; doi:10.7150/jca.94202)
Supplement: Supplementary file 1 — Supplementary figures. [file jcav15p3675s1.pdf]

Supplementary Materials:

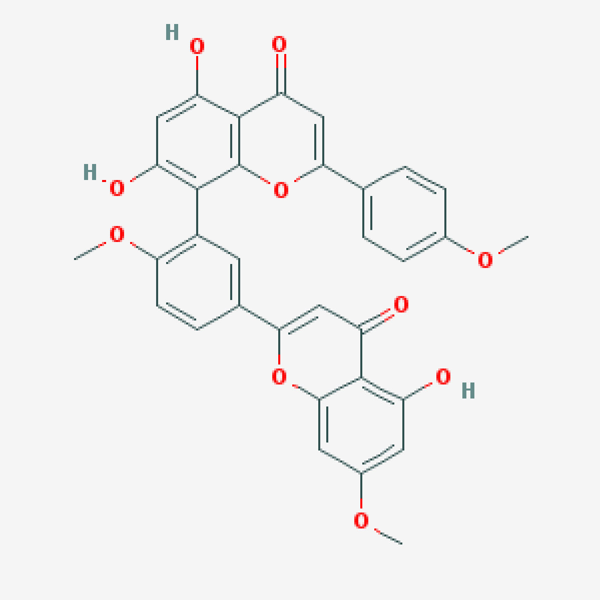

Figure S1. The molecular structure of sciadoptitysin.

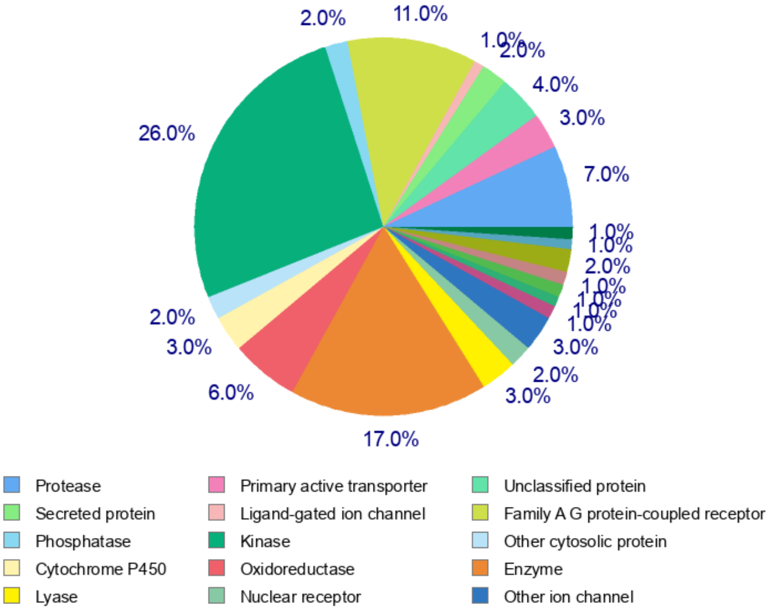

Figure S2. Classes of sciadopitysin potential targets.
