# Supplementary material for: Network Pharmacology and Bioinformatics Analysis to Identify the Molecular Targets and its Biological Mechanisms of Sciadopitysin against Glioblastoma
Source: J Cancer. 2024 May 13;15(12):3675–83. doi: 10.7150/jca.94202 (PMC11190769; doi:10.7150/jca.94202)
Supplement: Supplementary file 2 — Raw data. [file jcav15p3675s2.zip › RawData/Figure 1 rawdata/SwissTargetPrediction.pdf]

SwissTargetPrediction

Target	Common name	Uniprot ID	ChEMBL ID	Target Class	Probability*	Known actives (3D/2D)
Beta-secretase 1	BACE1	P56817	CHEMBL4822	Protease	0.880904793822	11 / 18
Protein-tyrosine phosphatase 1B	PTPN1	P18031	CHEMBL335	Phosphatase	0.229011931476	18 / 17
Transitional endoplasmic reticulum ATPase	VCP	P55072	CHEMBL1075145	Primary active transporter	0.171503305832	2 / 1
Placenta growth factor	PGF	P49763	CHEMBL1697671	Unclassified protein	0.142773751522	0 / 1
Vascular endothelial growth factor A	VEGFA	P15692	CHEMBL1783	Secreted protein	0.142773751522	0 / 1
GABA-A receptor; alpha-1/beta-2/ gamma-2	GABRA1 GABRB2 GABRG2	P14867 P47870 P18507	CHEMBL2095172	Ligand-gated ion channel	0.142773751522	0 / 1
Serotonin 2c (5-HT2c) receptor	HTR2C	P28335	CHEMBL225	Family A G protein-coupled receptor	0.142773751522	0 / 2
Delta opioid receptor	OPRD1	P41143	CHEMBL236	Family A G protein-coupled receptor	0.133198489461	3 / 5
Dopamine D3 receptor	DRD3	P35462	CHEMBL234	Family A G protein-coupled receptor	0.0853031603775	4 / 1
Cyclin-dependent kinase 5/CDK5 activator 1	CDK5R1 CDK5	Q15078 Q00535	CHEMBL1907600	Kinase	0.0757082396102	0 / 17
ATP-binding cassette sub-family G member 2	ABCG2	Q9UNQ0	CHEMBL5393	Primary active transporter	0.0661303830337	1 / 50
Induced myeloid leukemia cell differentiation protein Mcl-1	MCL1	Q07820	CHEMBL4361	Other cytosolic protein	0.0661303830337	9 / 4
Adenosine A1 receptor (by homology)	ADORA1	P30542	CHEMBL226	Family A G protein-coupled receptor	0.0661303830337	0 / 23
Adenosine A3 receptor	ADORA3	P0DMS8	CHEMBL256	Family A G protein-coupled receptor	0.0661303830337	0 / 18
Cytochrome P450 1B1	CYP1B1	Q16678	CHEMBL4878	Cytochrome P450	0.0661303830337	0 / 45
P-glycoprotein 1	ABCB1	P08183	CHEMBL4302	Primary active transporter	0.0661303830337	7 / 47
Aldose reductase	AKR1B1	P15121	CHEMBL1900	Enzyme	0.0661303830337	7 / 66
NEDD8-activating enzyme E1 regulatory subunit	NAE1	Q13564	CHEMBL2016431	Unclassified protein	0.0661303830337	0 / 1
Protein kinase C beta	PRKCB	P05771	CHEMBL3045	Kinase	0.0661303830337	69 / 0
Cytochrome P450 19A1	CYP19A1	P11511	CHEMBL1978	Cytochrome P450	0.0661303830337	5 / 18
Acetylcholinesterase	ACHE	P22303	CHEMBL220	Hydrolase	0.0661303830337	4 / 45
Adenosine A2a	ADORA2A	P29274	CHEMBL251	Family A G	0.0661303830337	0 / 11

Target	Common name	Uniprot ID	ChEMBL ID	Target Class	Probability*	Known actives (3D/2D)
receptor (by homology)				protein-coupled receptor		
NADPH oxidase 4	NOX4	Q9NPH5	CHEMBL1250375	Enzyme	0.0661303830337	0 / 8
Xanthine dehydrogenase	XDH	P47989	CHEMBL1929	Oxidoreductase	0.0661303830337	0 / 20
Lysine-specific histone demethylase 1	KDM1A	O60341	CHEMBL6136	Eraser	0.0661303830337	1 / 0
Nuclear factor NF-kappa-B p65 subunit	RELA	Q04206	CHEMBL5533	Transcription factor	0.0661303830337	3 / 0
Cyclooxygenase-2	PTGS2	P35354	CHEMBL230	Oxidoreductase	0.0661303830337	1 / 13
Tankyrase-2	TNKS2	Q9H2K2	CHEMBL6154	Enzyme	0.0661303830337	0 / 12
Tankyrase-1	TNKS	O95271	CHEMBL6164	Enzyme	0.0661303830337	0 / 26
Estrogen receptor alpha	ESR1	P03372	CHEMBL206	Nuclear receptor	0.0661303830337	4 / 20
Protein kinase C gamma	PRKCG	P05129	CHEMBL2938	Kinase	0.0661303830337	43 / 0
Multidrug resistance-associated protein 1	ABCC1	P33527	CHEMBL3004	Primary active transporter	0.0661303830337	0 / 11
Ornithine decarboxylase	ODC1	P11926	CHEMBL1869	Lyase	0.0661303830337	0 / 1
L-lactate dehydrogenase A chain	LDHA	P00338	CHEMBL4835	Enzyme	0.0661303830337	2 / 0
L-lactate dehydrogenase B chain	LDHB	P07195	CHEMBL4940	Enzyme	0.0661303830337	1 / 0
Troponin, cardiac muscle	TNNC1<br>TNNT2<br>TNNI3	P63316<br>P45379<br>P19429	CHEMBL2095202	Unclassified protein	0.0661303830337	2 / 0
Isocitrate dehydrogenase [NADP] cytoplasmic	IDH1	O75874	CHEMBL2007625	Enzyme	0.0661303830337	2 / 0
Heat shock protein HSP 90-alpha	HSP90AA1	P07900	CHEMBL3880	Other cytosolic protein	0.0661303830337	24 / 0
Fatty acid synthase	FASN	P49327	CHEMBL4158	Transferase	0.0661303830337	3 / 0
Butyrylcholinesterase	BCHE	P06276	CHEMBL1914	Hydrolase	0.0661303830337	4 / 7
Monoamine oxidase A	MAOA	P21397	CHEMBL1951	Oxidoreductase	0.0661303830337	0 / 5
Cyclin-dependent kinase 1/cyclin B	CCNB3<br>CDK1<br>CCNB1<br>CCNB2	Q8WWL7<br>P06493<br>P14635<br>O95067	CHEMBL2094127	Other cytosolic protein	0.0661303830337	0 / 7
Estrogen receptor beta	ESR2	Q92731	CHEMBL242	Nuclear receptor	0.0661303830337	0 / 18
Cyclin-dependent kinase 6	CDK6	Q00534	CHEMBL2508	Kinase	0.0661303830337	0 / 4
Transthyretin	TTR	P02766	CHEMBL3194	Secreted protein	0.0661303830337	0 / 2
Casein kinase II alpha	CSNK2A1	P68400	CHEMBL3629	Kinase	0.0661303830337	0 / 2
Aldo-keto reductase family 1 member B10	AKR1B10	O60218	CHEMBL5983	Enzyme	0.0661303830337	0 / 3
DNA-3-methyladenine	MPG	P29372	CHEMBL3396943	Enzyme	0.0661303830337	1 / 1

Target	Common name	Uniprot ID	ChEMBL ID	Target Class	Probability*	Known actives (3D/2D)
glycosylase						
Maternal embryonic leucine zipper kinase	MELK	Q14680	CHEMBL4578	Kinase	0.0661303830337	269 / 0
Carbonic anhydrase II	CA2	P00918	CHEMBL205	Lyase	0.0661303830337	10 / 15
Carbonic anhydrase XII	CA12	O43570	CHEMBL3242	Lyase	0.0661303830337	10 / 14
HERG	KCNH2	Q12809	CHEMBL240	Voltage-gated ion channel	0.0661303830337	1 / 0
C-C chemokine receptor type 1	CCR1	P32246	CHEMBL2413	Family A G protein-coupled receptor	0.0661303830337	1 / 0
Tyrosinase	TYR	P14679	CHEMBL1973	Oxidoreductase	0.0661303830337	0 / 2
Aryl hydrocarbon receptor	AHR	P35869	CHEMBL3201	Transcription factor	0.0661303830337	0 / 1
Estrogen-related receptor alpha	ESRRA	P11474	CHEMBL3429	Nuclear receptor	0.0661303830337	0 / 2
Pyruvate dehydrogenase kinase isoform 1	PDK1	Q15118	CHEMBL4766	Kinase	0.0661303830337	48 / 0
Coagulation factor VII	F7	P08709	CHEMBL3991	Protease	0.0661303830337	3 / 0
Nitric oxide synthase, inducible (by homology)	NOS2	P35228	CHEMBL4481	Enzyme	0.0661303830337	5 / 3
Heat shock protein 75 kDa, mitochondrial	TRAP1	Q12931	CHEMBL1075132	Other cytosolic protein	0.0	2 / 0
Heat shock protein HSP 90-beta	HSP90AB1	P08238	CHEMBL4303	Other cytosolic protein	0.0	9 / 0
Carbonic anhydrase VII	CA7	P43166	CHEMBL2326	Lyase	0.0	3 / 11
Serine/threonine-protein kinase AKT	AKT1	P31749	CHEMBL4282	Kinase	0.0	2 / 3
Metastin receptor	KISS1R	Q969F8	CHEMBL5413	Family A G protein-coupled receptor	0.0	21 / 0
Receptor-type tyrosine-protein phosphatase S	PTPRS	Q13332	CHEMBL2396508	Phosphatase	0.0	0 / 8
AMY1C	AMY1A	P04745	CHEMBL2478	Enzyme	0.0	0 / 1
G protein-coupled receptor kinase 6	GRK6	P43250	CHEMBL6144	Kinase	0.0	0 / 3
Tyrosine-protein kinase SRC	SRC	P12931	CHEMBL267	Kinase	0.0	9 / 9
G-protein coupled receptor 35	GPR35	Q9HC97	CHEMBL1293267	Family A G protein-coupled receptor	0.0	0 / 4
Death-associated protein kinase 1	DAPK1	P53355	CHEMBL2558	Kinase	0.0	0 / 2
Solute carrier family 22 member 12	SLC22A12	Q96S37	CHEMBL6120	Electrochemical transporter	0.0	0 / 1
Serine/threonine-protein kinase mTOR	MTOR	P42345	CHEMBL2842	Kinase	0.0	1 / 0
PI3-kinase p110-alpha	PIK3CA	P42336	CHEMBL4005	Enzyme	0.0	1 / 0

Target	Common name	Uniprot ID	ChEMBL ID	Target Class	Probability*	Known actives (3D/2D)
subunit						
Serine/threonine-protein kinase Chk1	CHEK1	O14757	CHEMBL4630	Kinase	0.0	13 / 0
Serine/threonine-protein kinase WEE1	WEE1	P30291	CHEMBL5491	Kinase	0.0	9 / 0
Carbonic anhydrase I	CA1	P00915	CHEMBL261	Lyase	0.0	10 / 9
Carbonic anhydrase IX	CA9	Q16790	CHEMBL3594	Lyase	0.0	11 / 5
Plasminogen	PLG	P00747	CHEMBL1801	Protease	0.0	1 / 3
Glucagon receptor	GCGR	P47871	CHEMBL1985	Family B G protein-coupled receptor	0.0	39 / 0
C-C chemokine receptor type 4	CCR4	P51679	CHEMBL2414	Family A G protein-coupled receptor	0.0	8 / 0
Histone deacetylase 1	HDAC1	Q13547	CHEMBL325	Eraser	0.0	3 / 0
Kappa Opioid receptor (by homology)	OPRK1	P41145	CHEMBL237	Family A G protein-coupled receptor	0.0	1 / 0
Androgen Receptor	AR	P10275	CHEMBL1871	Nuclear receptor	0.0	0 / 14
Carbonyl reductase [NADPH] 1	CBR1	P16152	CHEMBL5586	Enzyme	0.0	0 / 2
Endoplasmin	HSP90B1	P14625	CHEMBL1075323	Other membrane protein	0.0	15 / 0
Plasma kallikrein	KLKB1	P03952	CHEMBL2000	Protease	0.0	2 / 0
Interleukin-8 receptor B	CXCR2	P25025	CHEMBL2434	Family A G protein-coupled receptor	0.0	6 / 0
Kallikrein 1	KLK1	P06870	CHEMBL2319	Protease	0.0	1 / 0
Kallikrein 2	KLK2	P20151	CHEMBL2442	Protease	0.0	1 / 0
Arachidonate 15-lipoxygenase	ALOX15	P16050	CHEMBL2903	Enzyme	0.0	5 / 7
Arachidonate 12-lipoxygenase	ALOX12	P18054	CHEMBL3687	Enzyme	0.0	3 / 9
Prostanoid EP3 receptor	PTGER3	P43115	CHEMBL3710	Family A G protein-coupled receptor	0.0	2 / 0
Poly [ADP-ribose] polymerase-1	PARP1	P09874	CHEMBL3105	Enzyme	0.0	0 / 9
Matrix metalloproteinase 9	MMP9	P14780	CHEMBL321	Protease	0.0	0 / 2
Matrix metalloproteinase 2	MMP2	P08253	CHEMBL333	Protease	0.0	0 / 2
Matrix metalloproteinase 12	MMP12	P39900	CHEMBL4393	Protease	0.0	0 / 1
Lymphocyte differentiation antigen CD38	CD38	P28907	CHEMBL4660	Enzyme	0.0	0 / 2
DNA topoisomerase I (by homology)	TOP1	P11387	CHEMBL1781	Isomerase	0.0	0 / 1

Target	Common name	Uniprot ID	ChEMBL ID	Target Class	Probability*	Known actives (3D/2D)
Arginase-1 (by homology)	ARG1	P05089	CHEMBL1075097	Enzyme	0.0	0 / 2
Protein kinase C iota	PRKCI	P41743	CHEMBL2598	Kinase	0.0	1 / 0
