# Supplementary material for: Network Pharmacology and Bioinformatics Analysis to Identify the Molecular Targets and its Biological Mechanisms of Sciadopitysin against Glioblastoma
Source: J Cancer. 2024 May 13;15(12):3675–83. doi: 10.7150/jca.94202 (PMC11190769; doi:10.7150/jca.94202)
Supplement: Supplementary file 2 — Raw data. [file jcav15p3675s2.zip › RawData/Figure 6 rawdata/Flow cytometry-Layout.pdf]

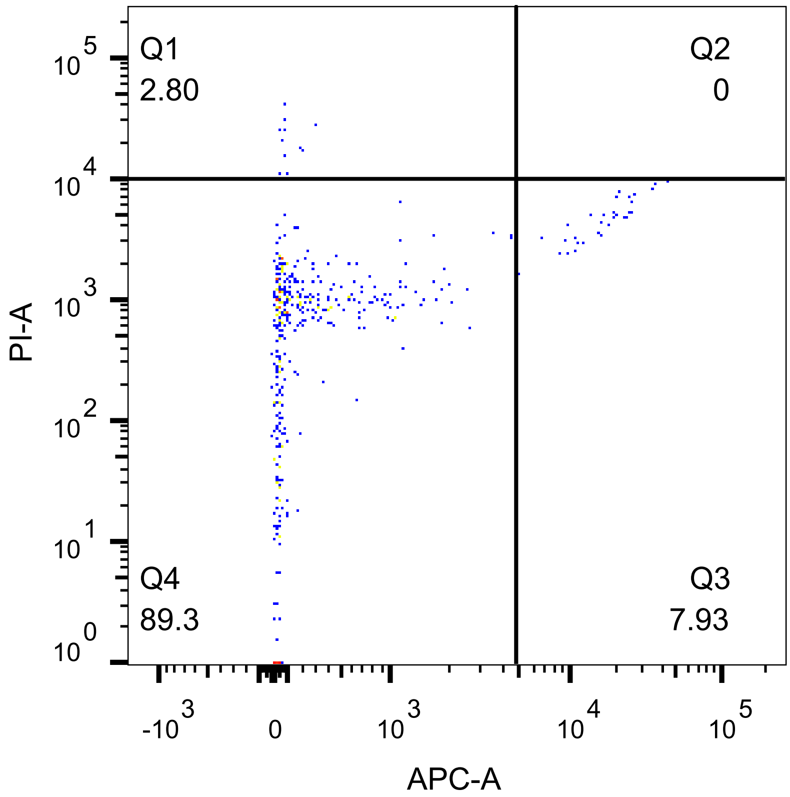


Compensation Controls_APC Stained Control.fcs
tumor cell
429

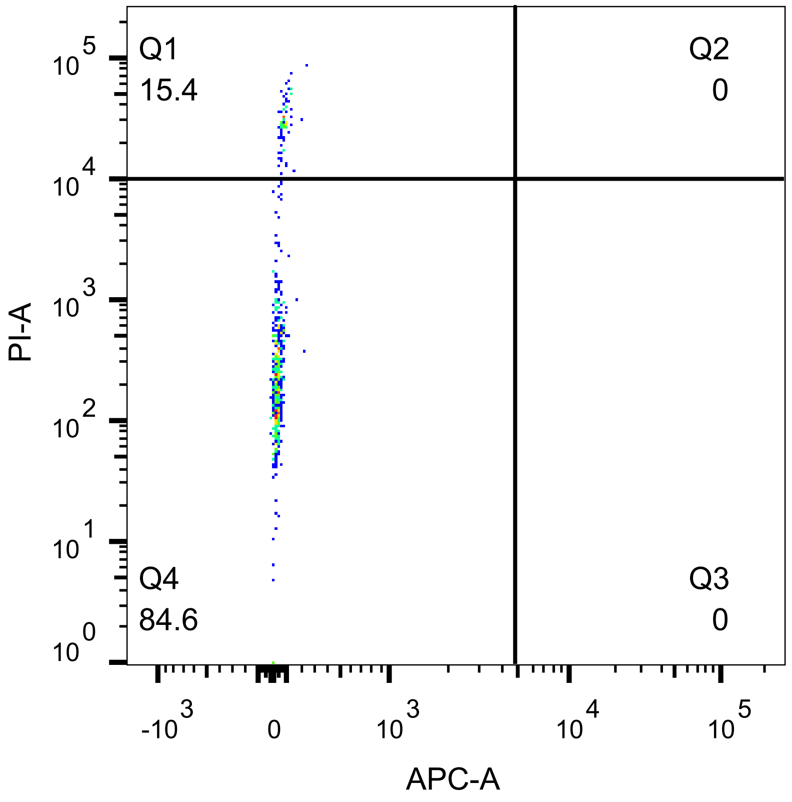


Compensation Controls_PI Stained Control.fcs
tumor cell
526

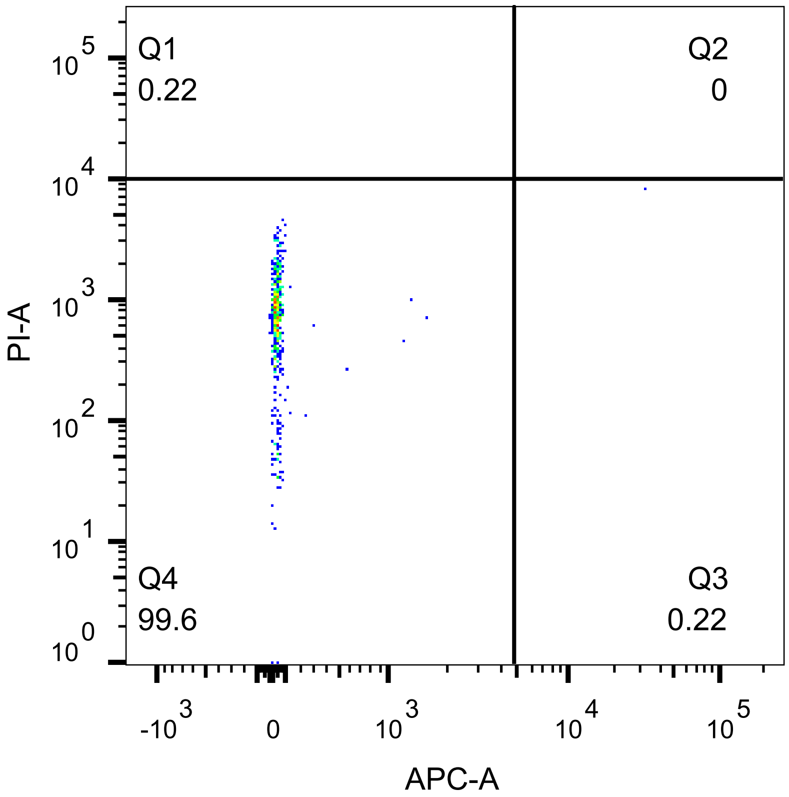


Compensation Controls_Unstained Control.fcs
tumor cell
464

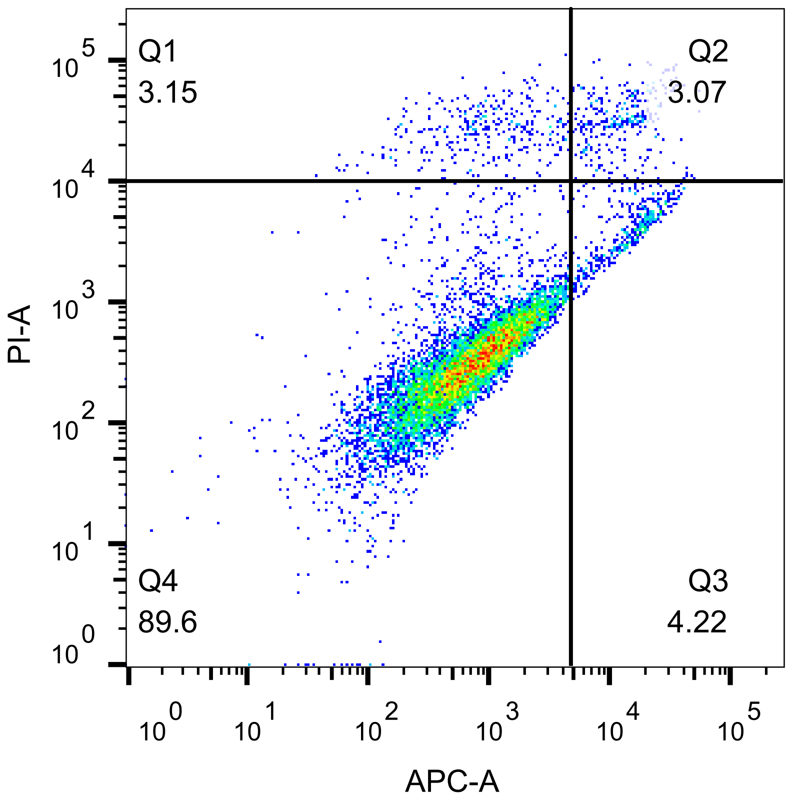


Specimen_001_Tube_001.fcs
tumor cell
10225

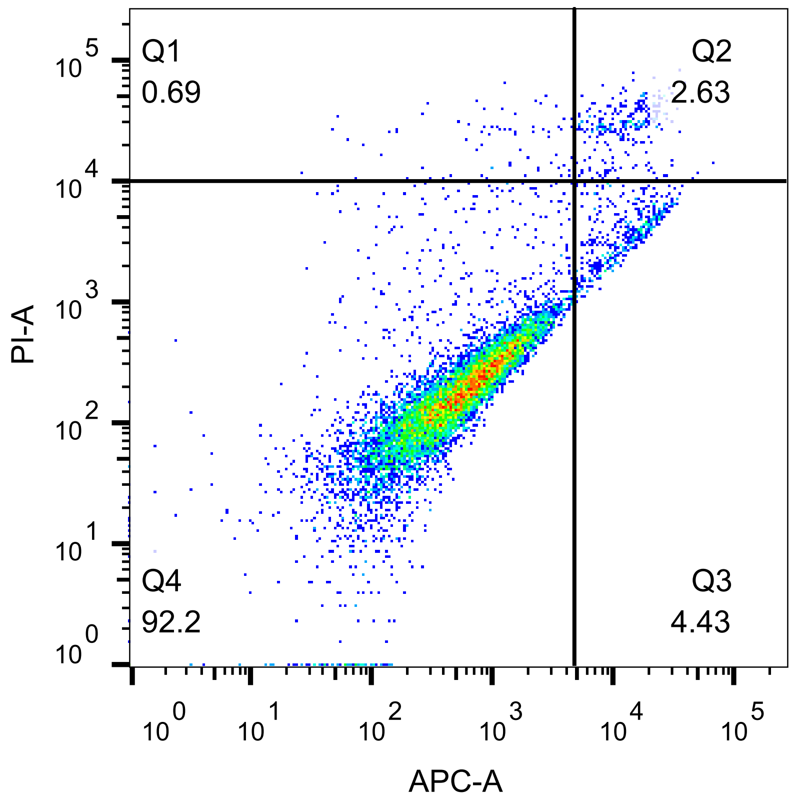


Specimen_001_Tube_002.fcs
tumor cell
10254

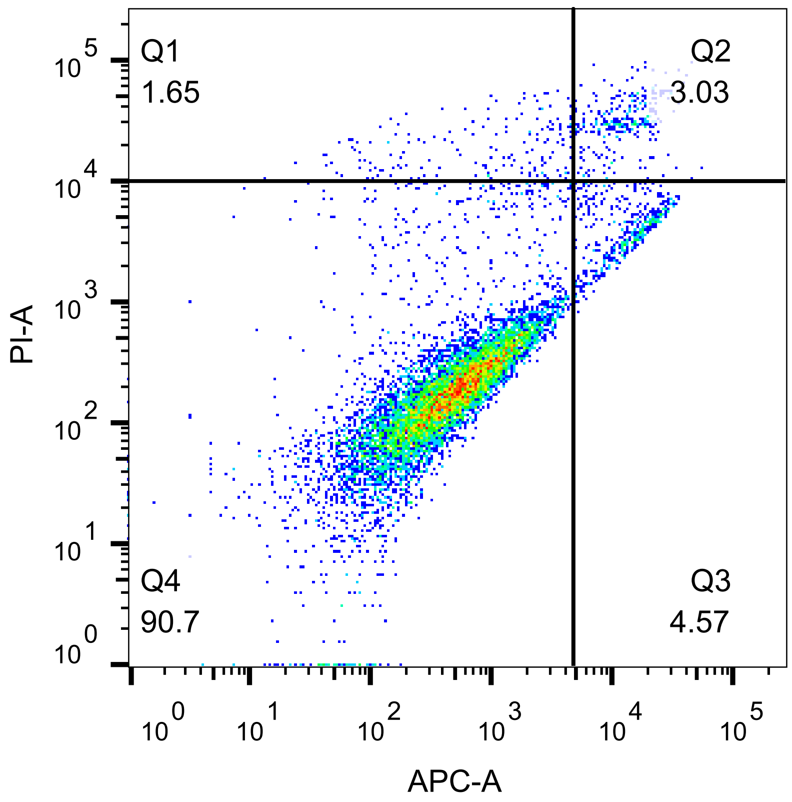


Specimen_001_Tube_003.fcs
tumor cell
10170

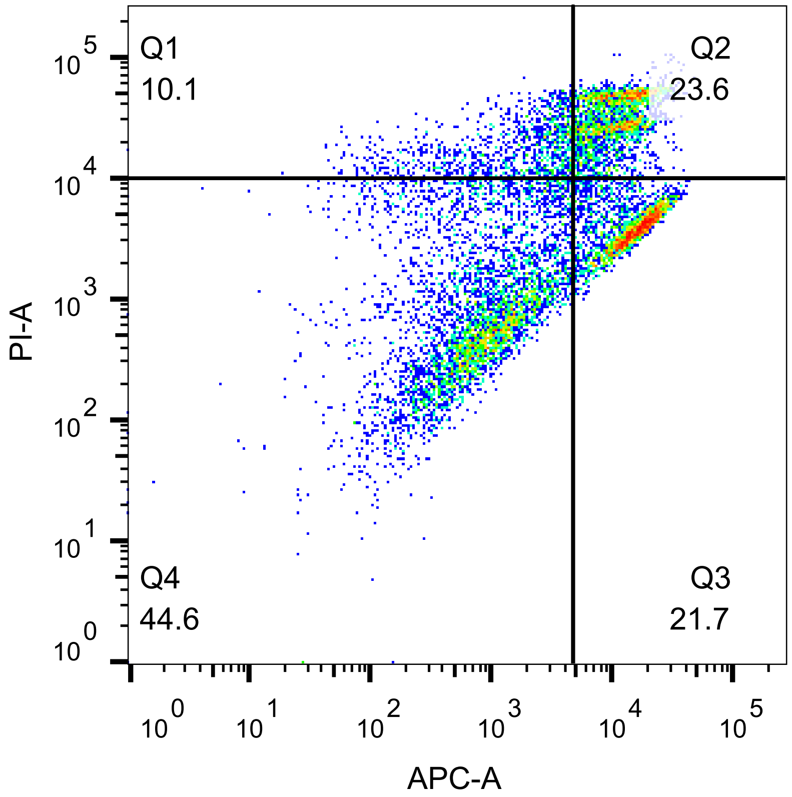


Specimen_001_Tube_004.fcs
tumor cell
10365

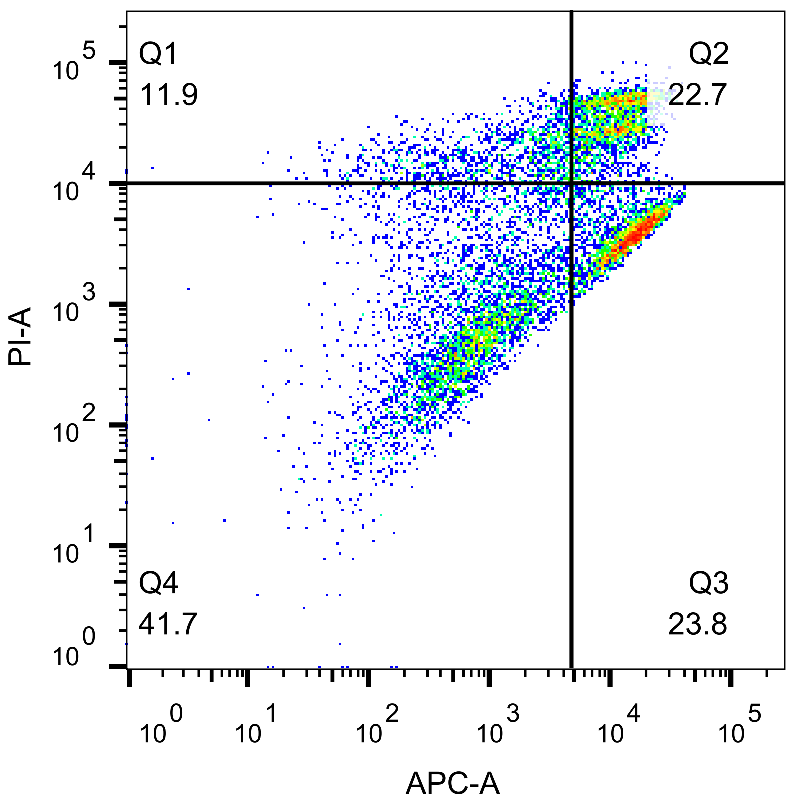


Specimen_001_Tube_005.fcs
tumor cell
10403

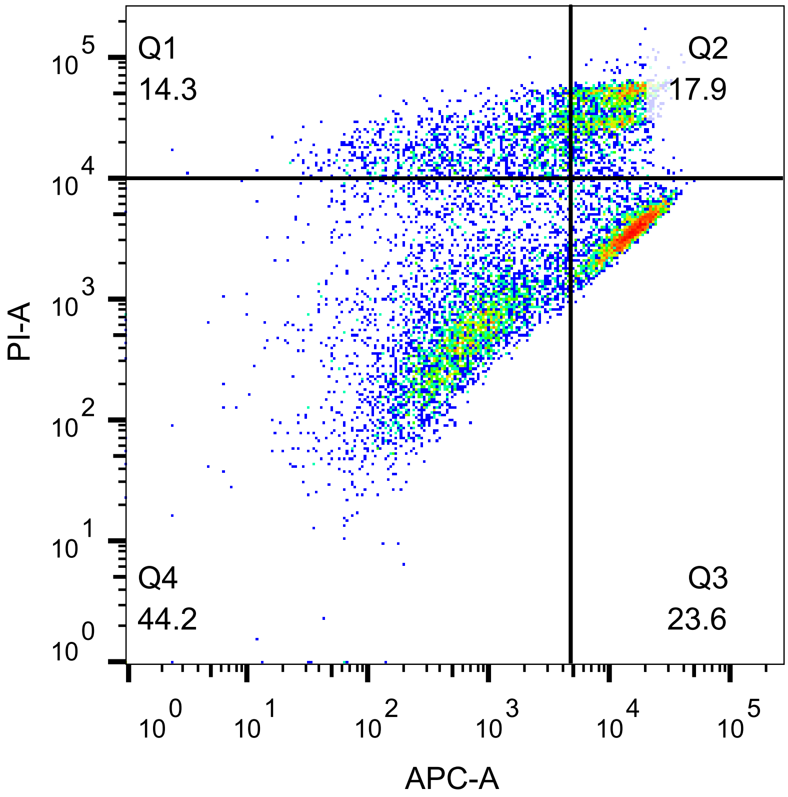


Specimen_001_Tube_006.fcs
tumor cell
10404
