# Supplementary material for: Network Pharmacology and Bioinformatics Analysis to Identify the Molecular Targets and its Biological Mechanisms of Sciadopitysin against Glioblastoma
Source: J Cancer. 2024 May 13;15(12):3675–83. doi: 10.7150/jca.94202 (PMC11190769; doi:10.7150/jca.94202)
Supplement: Supplementary file 2 — Raw data. [file jcav15p3675s2.zip › RawData/Figure 6 rawdata/Original Images for BlotsGels.pdf]

HSP90α

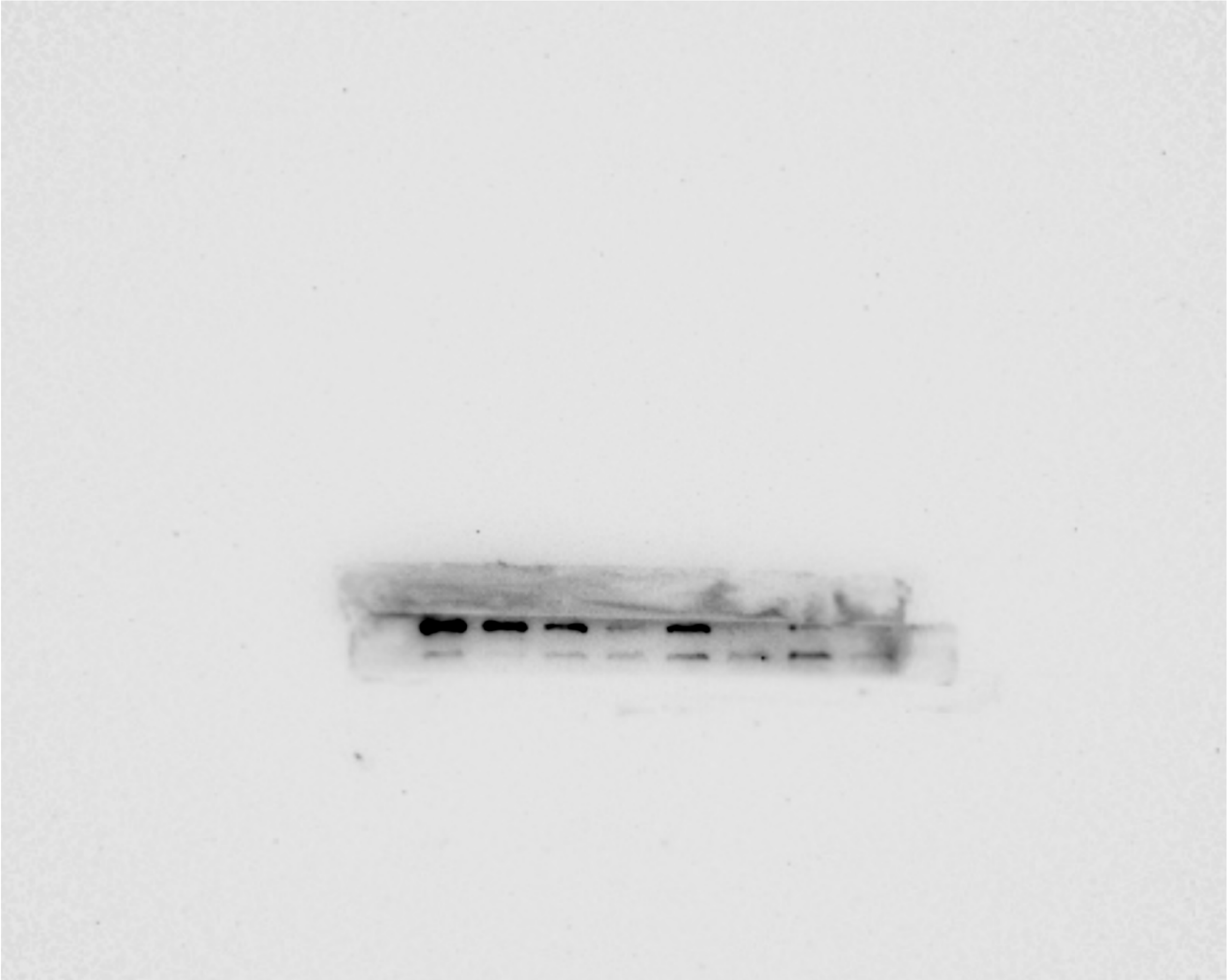

AKT1

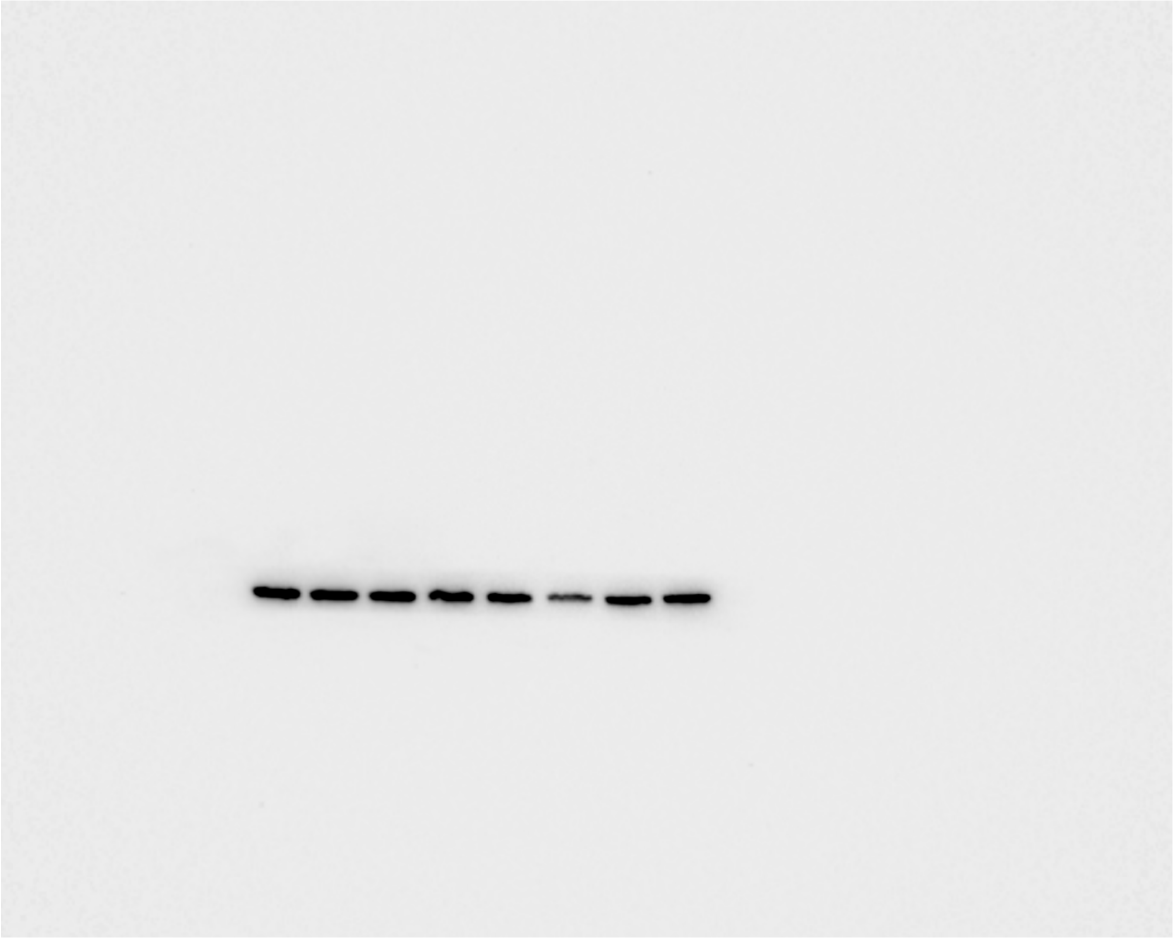

Actin

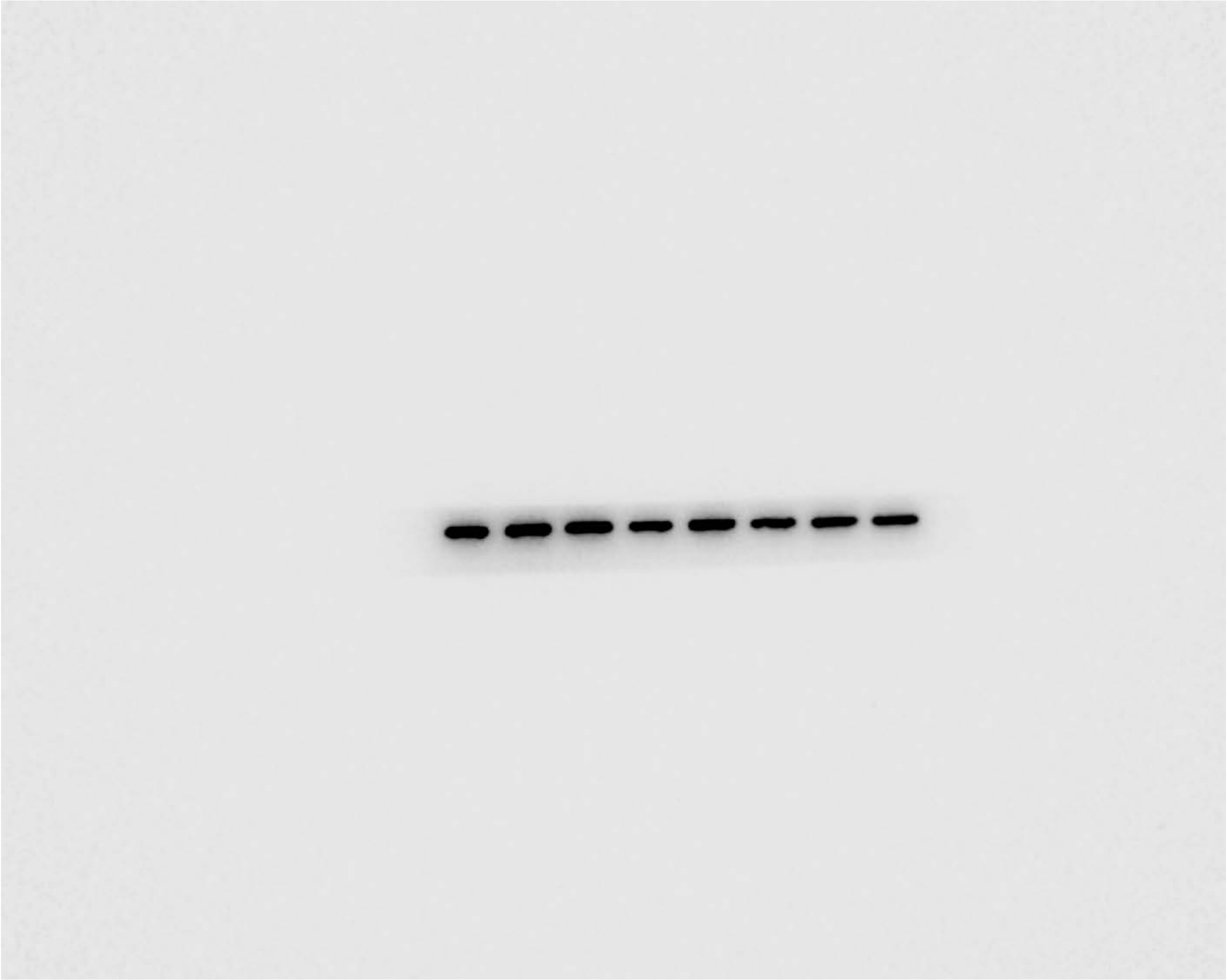

Note:

Lane 1 : Control 1

Lane 2: sciadopitysin 100μM treatment 1

Lane 3 : Control 2

Lane 4: sciadopitysin 100μM treatment 2

Lane 5 : Control 3

Lane 6: sciadopitysin 100μM treatment 3

Lane 7: other samples

Lane 8: other samples
