# Supplementary figures and images for: Network Pharmacology and Bioinformatics Analysis to Identify the Molecular Targets and its Biological Mechanisms of Sciadopitysin against Glioblastoma
Source: J Cancer. 2024 May 13;15(12):3675–83. doi: 10.7150/jca.94202 (PMC11190769; doi:10.7150/jca.94202)

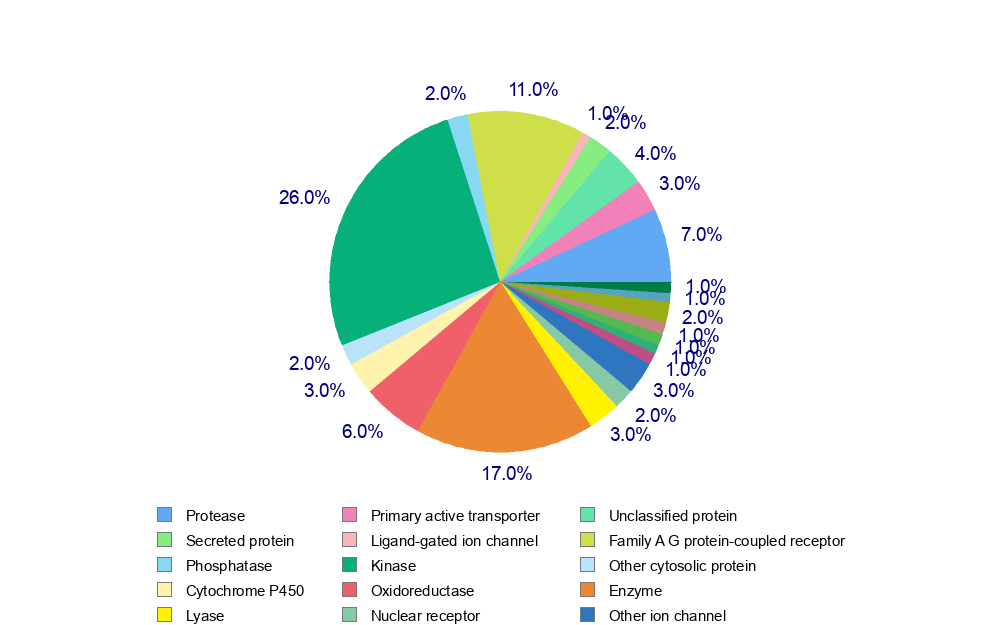

Supplement: Supplementary file 2 — Raw data. [file jcav15p3675s2.zip › RawData/Figure 1 rawdata/pieChartTopAll.png]

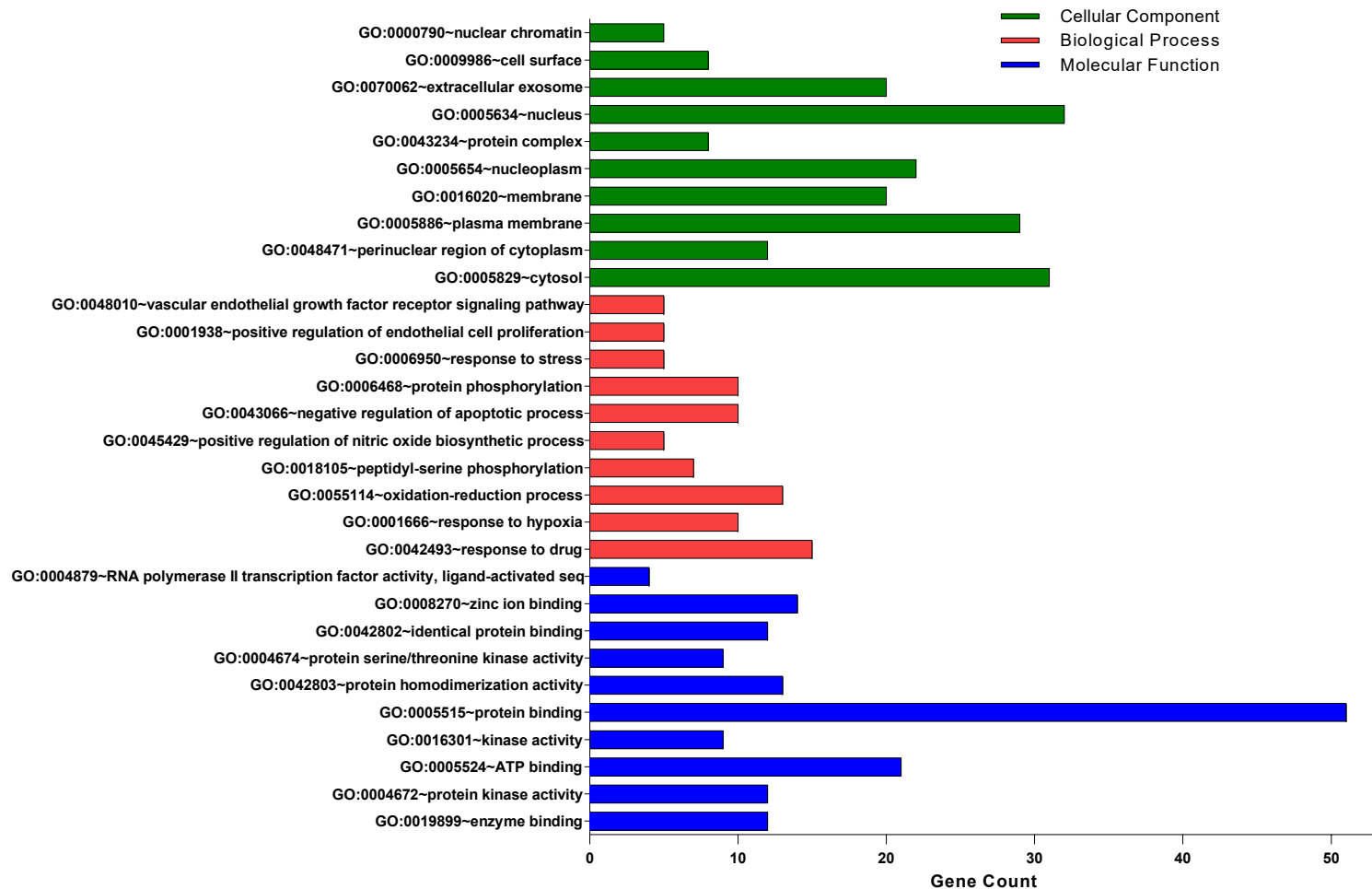

Supplement: Supplementary file 2 — Raw data. [file jcav15p3675s2.zip › RawData/Figure 2 rawdata/GO analysis results.pdf]

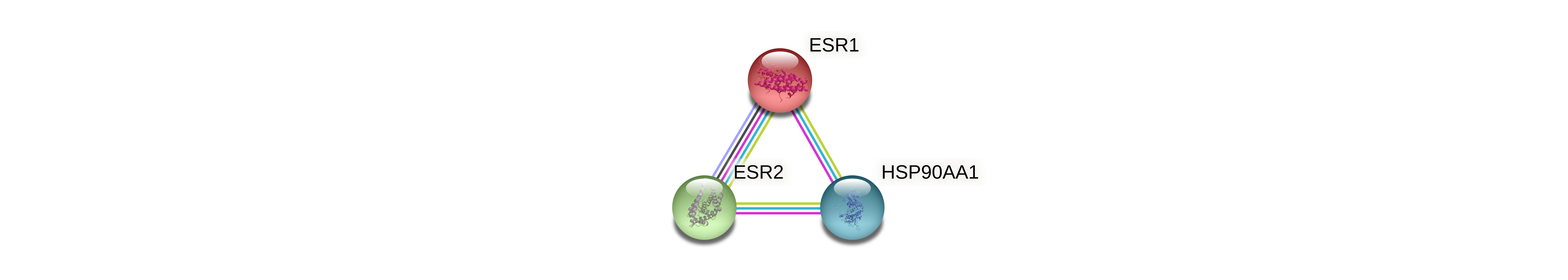

Supplement: Supplementary file 2 — Raw data. [file jcav15p3675s2.zip › RawData/Figure 3 rawdata/Fig 3b/ESR-mediated signaling string_hires_image.png]

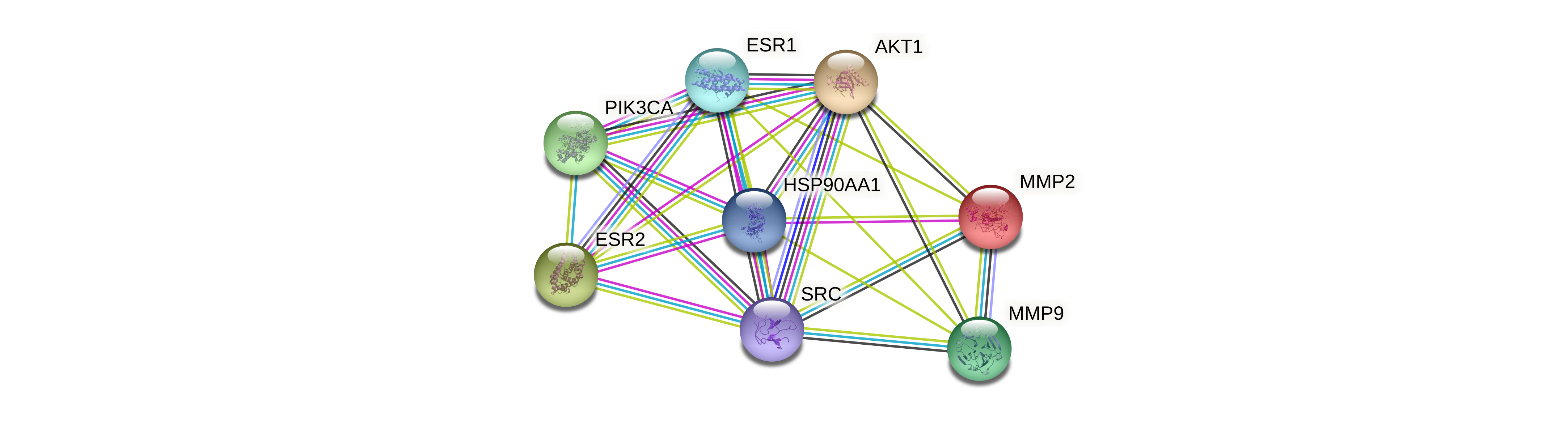

Supplement: Supplementary file 2 — Raw data. [file jcav15p3675s2.zip › RawData/Figure 3 rawdata/Fig 3b/Extra-nuclear estrogen signaling string_hires_image.png]

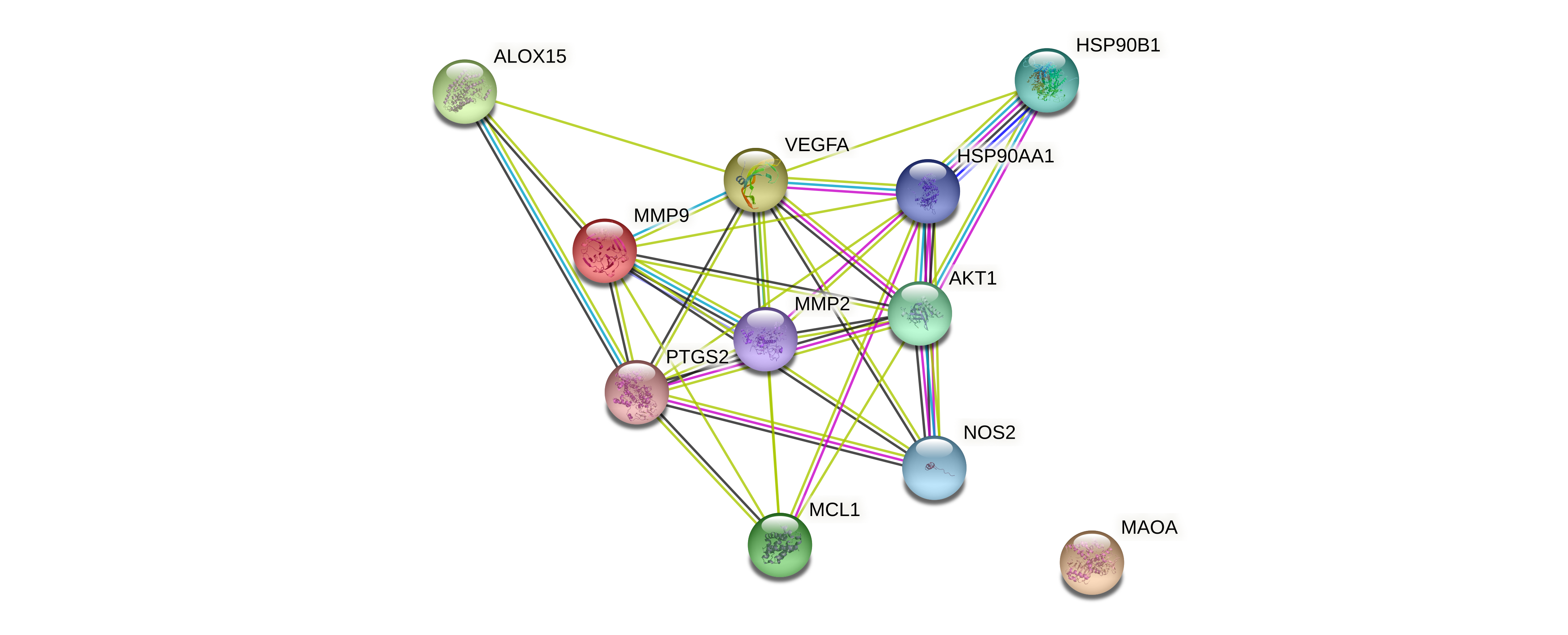

Supplement: Supplementary file 2 — Raw data. [file jcav15p3675s2.zip › RawData/Figure 3 rawdata/Fig 3b/Interleukin-4 and Interleukin-13 signaling string_hires_image.png]

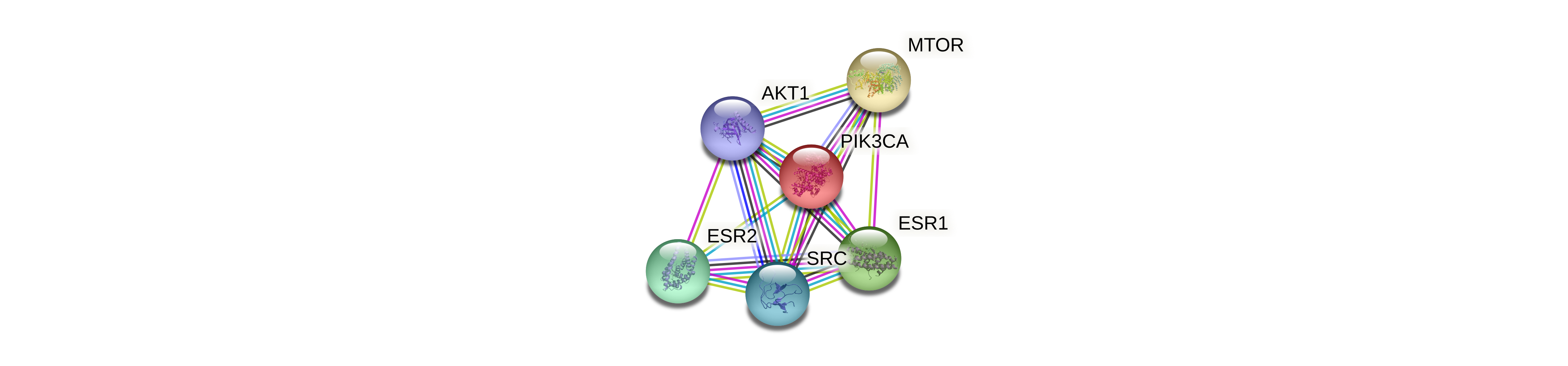

Supplement: Supplementary file 2 — Raw data. [file jcav15p3675s2.zip › RawData/Figure 3 rawdata/Fig 3b/PIP3 activates AKT signaling string_hires_image.png]

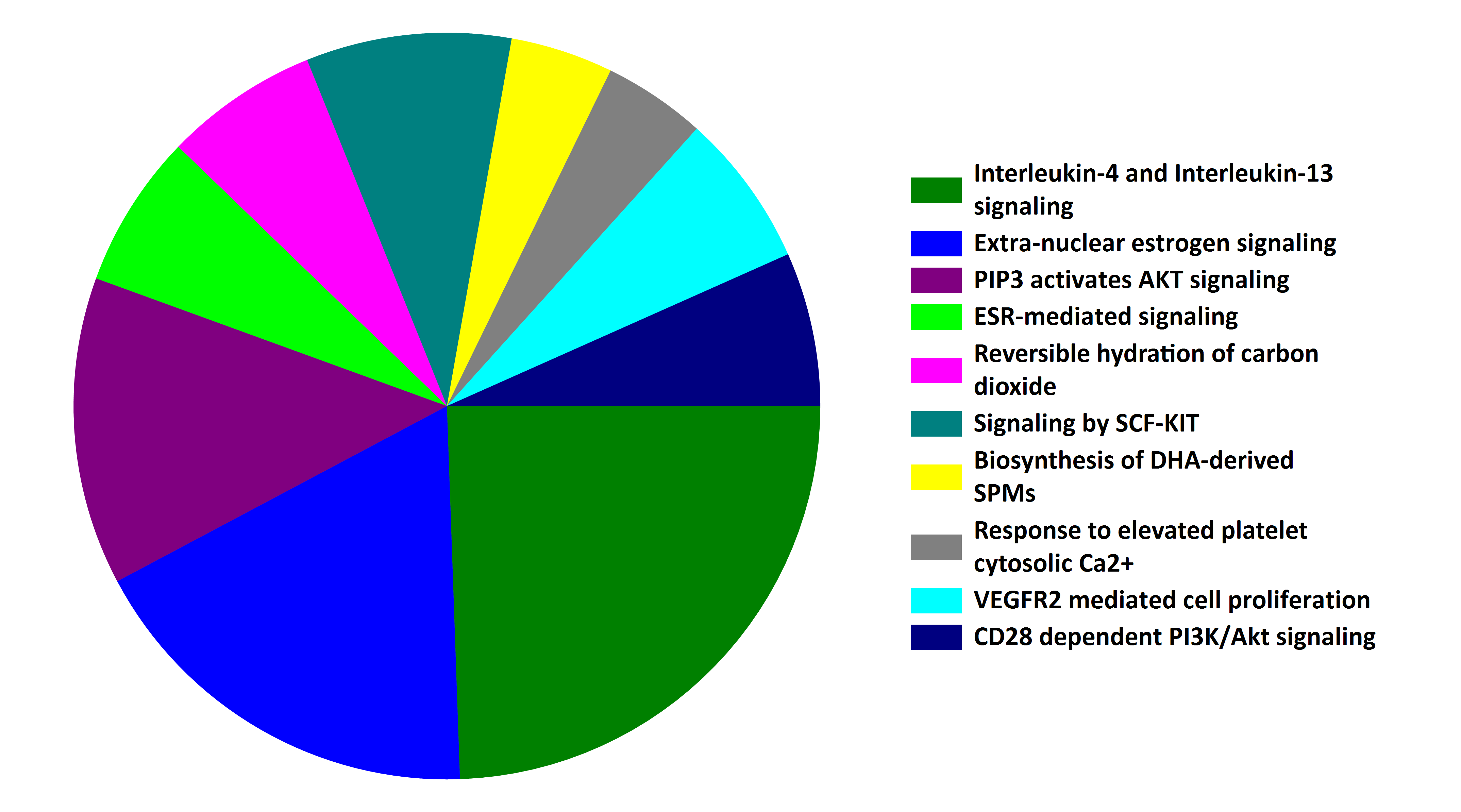

Supplement: Supplementary file 2 — Raw data. [file jcav15p3675s2.zip › RawData/Figure 3 rawdata/Signaling enrichment Chart.tiff]

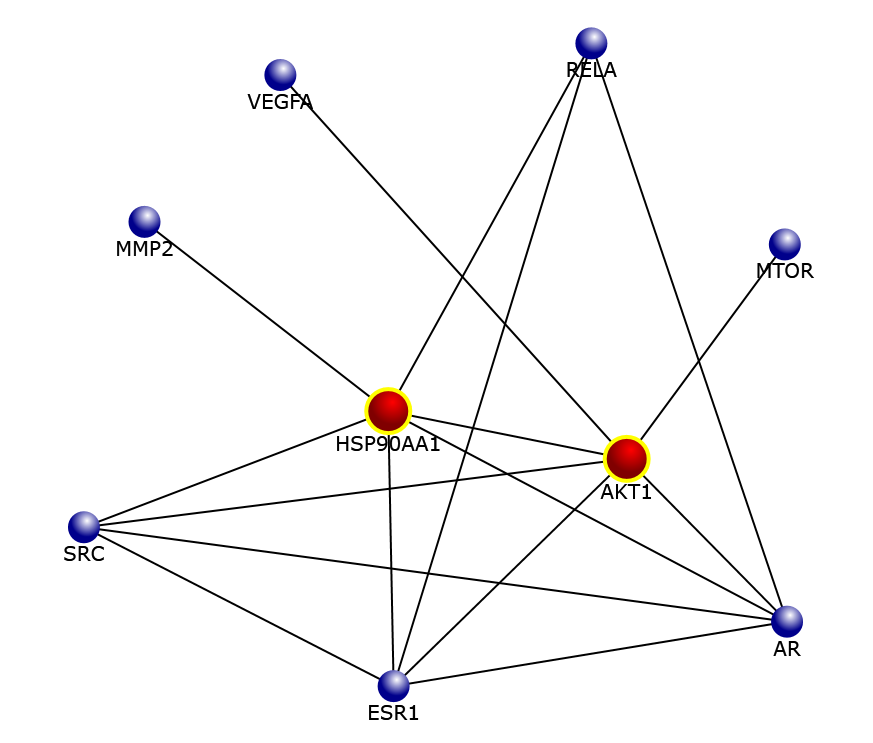

Supplement: Supplementary file 2 — Raw data. [file jcav15p3675s2.zip › RawData/Figure 4 rawdata/InteractionDiagram after CFinder k12 Funrich one core clusters tif.tiff]

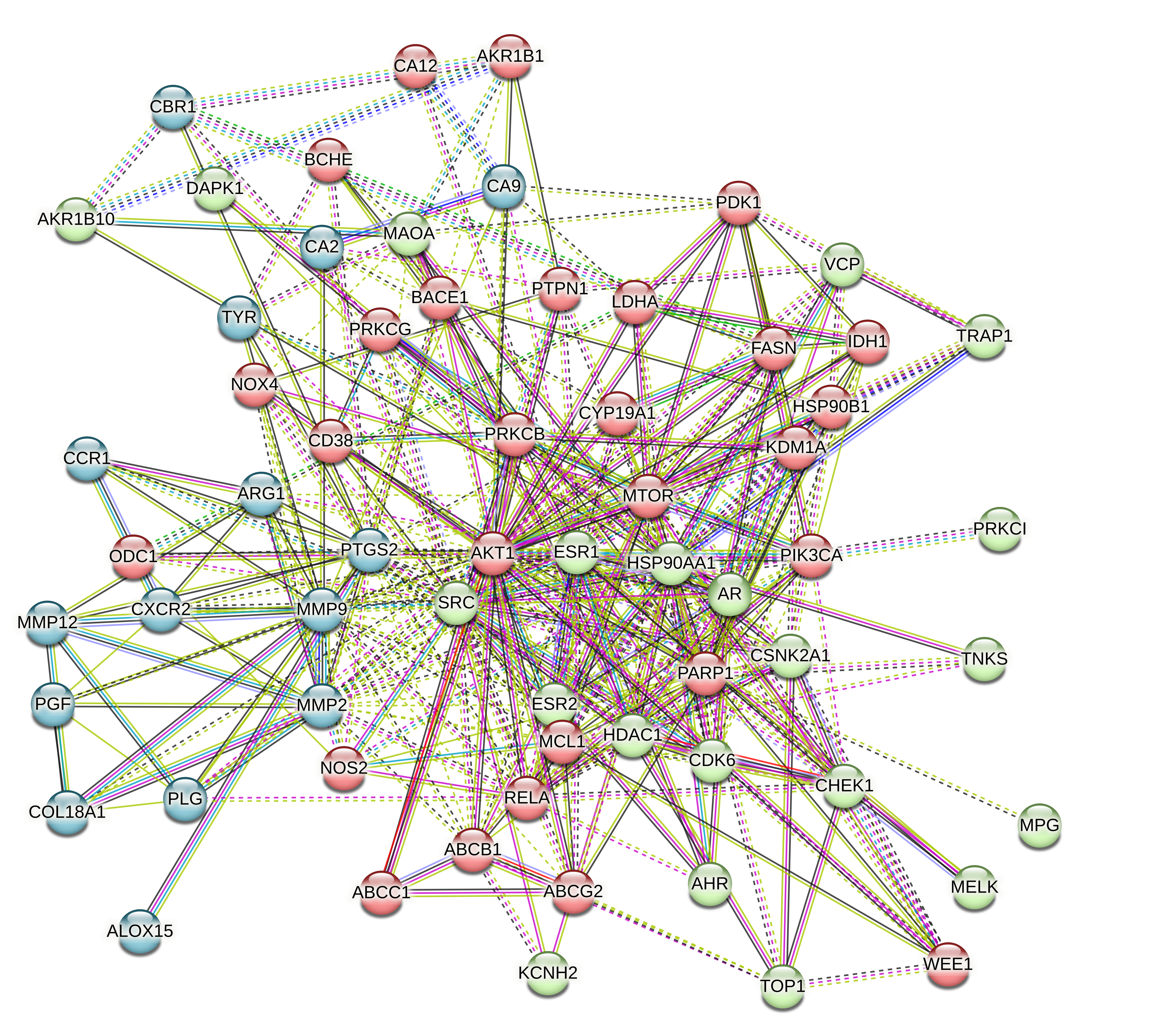

Supplement: Supplementary file 2 — Raw data. [file jcav15p3675s2.zip › RawData/Figure 4 rawdata/string_hires_image.png]
